# Isolation of a Bacteriophage Specific for a New Capsular Type of *Klebsiella pneumoniae* and Characterization of Its Polysaccharide Depolymerase

**DOI:** 10.1371/journal.pone.0070092

**Published:** 2013-08-02

**Authors:** Chun-Ru Hsu, Tzu-Lung Lin, Yi-Jiun Pan, Pei-Fang Hsieh, Jin-Town Wang

**Affiliations:** 1 Department of Microbiology, National Taiwan University College of Medicine, Taipei, Taiwan; 2 Department of Internal Medicine, National Taiwan University Hospital, Taipei, Taiwan; Naval Research Laboratory, United States of America

## Abstract

**Background:**

*Klebsiella pneumoniae* is one of the major pathogens causing hospital-acquired multidrug-resistant infections. The capsular polysaccharide (CPS) is an important virulence factor of *K. pneumoniae*. With 78 capsular types discovered thus far, an association between capsular type and the pathogenicity of *K. pneumoniae* has been observed.

**Methodology/Principal Findings:**

To investigate an initially non-typeable *K. pneumoniae* UTI isolate NTUH-K1790N, the *cps* gene region was sequenced. By NTUH-K1790N *cps*-PCR genotyping, serotyping and determination using a newly isolated capsular type-specific bacteriophage, we found that NTUH-K1790N and three other isolates Ca0507, Ca0421 and C1975 possessed a new capsular type, which we named KN2. Analysis of a KN2 CPS^−^ mutant confirmed the role of capsule as the target recognized by the antiserum and the phage. A newly described lytic phage specific for KN2 *K. pneumoniae*, named 0507-KN2-1, was isolated and characterized using transmission electron microscopy. Whole-genome sequencing of 0507-KN2-1 revealed a 159 991 bp double-stranded DNA genome with a G+C content of 46.7% and at least 154 open reading frames. Based on its morphological and genomic characteristics, 0507-KN2-1 was classified as a member of the *Myoviridae* phage family. Further analysis of this phage revealed a 3738-bp gene encoding a putative polysaccharide depolymerase. A recombinant form of this protein was produced and assayed to confirm its enzymatic activity and specificity to KN2 capsular polysaccharides. KN2 *K. pneumoniae* strains exhibited greater sensitivity to this depolymerase than these did to the cognate phage, as determined by spot analysis.

**Conclusions/Significance:**

Here we report that a group of clinical strains possess a novel *Klebsiella* capsular type. We identified a KN2-specific phage and its polysaccharide depolymerase, which could be used for efficient capsular typing. The lytic phage and depolymerase also have potential as alternative therapeutic agents to antibiotics for treating *K. pneumoniae* infections, especially against antibiotic-resistant strains.

## Introduction

*Klebsiella pneumoniae*, a Gram-negative enteric bacterium, is a common pathogen that causes hospital-acquired urinary tract infections (UTIs), septicemia, and pneumonia [1]. In the recent decades, community-acquired pyogenic liver abscess (PLA) caused by *K. pneumoniae* complicated with metastatic meningitis and endophthalmitis has emerged globally, especially in Asia [2]–[16]. The capsule is an important virulence factor in *K. pneumoniae*. With at least 78 capsular serotypes defined, associations of certain serotypes with specific *K. pneumoniae-*induced diseases have been observed [17], [18]. The majority of *K. pneumoniae* isolates causing liver abscesses belong to serotypes K1 and K2 [10], [19], the most virulent of the known serotypes (Mizuta, K., 1983). Our studies showed that *K. pneumoniae* strains causing PLA belonged to capsular types K1 (∼80%), K2, K5, K20, K54, K57, and a new type [9], [20]. In a collection from a medical center in Taiwan, K1 was found most frequently among bacteremic isolates, and K2 was found mostly in respiratory tract specimens [10]. Among *Klebsiella* bacteremic isolates in Europe and North America, capsular serotypes K2, K21, and K55 were reported to be the most common [21]. The distribution of *K. pneumoniae* capsular serotypes appears to differ worldwide [22]–[25].

The capsule of *KIebsiella* spp. can be typed using a variety of techniques [26]. Serological diagnosis is commonly used to determine *Klebsiella* capsular serotypes, mainly via recognition of CPS variants by specific antibodies. However, serotyping is usually expensive, and serological cross-reactions between two or more capsular serotypes are common in clinical isolates.

Molecular techniques for capsular typing circumvent these problems and have thus become the current trend. Polymerase chain reaction (PCR) has been used to accurately and rapidly type *Klebsiella* based on DNA sequence variations in the *capsular polysaccharide synthesis* (*cps*) gene cluster [5]. The chromosomal *cps* region usually consists of 17∼25 genes encoding proteins involved in the translocation and assembly of surface polysaccharides. However, *cps* genotyping requires DNA sequences that can be distinguished between different capsular types. To date, only a limited number of specific *cps* sequences are known. Typing by use of bacteriophages(phages) that infect bacteria is an attractive method to diagnostic laboratories because it is simple and rapid, allowing analysis of large numbers of isolates [27]. *Klebsiella* phages have been used to differentiate serologically cross-reactive strains [28] and for epidemiological typing [29]. However, phage typing requires the isolation of phages that have high specificity for particular capsular serotypes. Because each of these typing methods has limitations or drawbacks, investigators usually employ 2 or more approaches to improve their typing accuracy.

Typing method limitations preclude serotype determination for some clinical isolates. For example, an Australian survey using antisera for typing reported that out of 293 *K. pneumoniae* isolates, 88 (30%) could not be typed and 54 (18%) had a positive reaction for more than one capsular type [22]. While typing *K. pneumoniae* clinical isolates in our laboratory using serotyping and *cps*-PCR, we found that UTI strain NTUH-K1790N could not be typed. In this study, we initially sequenced the *cps* locus of this strain. We tried to characterize this unidentified capsular type using several typing methods.

## Methods

### Bacterial Strains

We purchased 77 *K. pneumoniae* capsular serotype (K) reference strains from Statens Serum Institute (Copenhagen, Denmark). In addition, strain A1142, identified in our previous study as a new serotype (KN1) [20], was used. In total, 265 *K. pneumoniae* clinical isolates were analyzed using *cps*-PCR, including: 124 strains collected from the National Taiwan University Hospital (32 blood isolates from patients with no tissue-invasive disease, 22 non-blood isolates from non-septic patients, and 70 UTI strains); 13 strains from En Chu Kong Hospital, Taiwan; 34 strains from Far Eastern Memorial Hospital, Taiwan; 15 strains from Hong Kong [7]; and 79 isolates from blood or cerebrospinal fluid (CSF) of patients obtained from the Department of Medical Microbiology, University of Manitoba, Winnipeg, Manitoba, Canada [20].

### Ethics Statement

The bacterial clinical strains used in this study were provided by collection of National Taiwan University Hospital, En Chu Kong Hospital, Far Eastern Memorial Hospital and University of Manitoba, described in previous publications [7], [20]. The Ethics Committee of National Taiwan University Hospital approved that no formal ethical approval was needed to use these bacterial strains, because the strains were remnant from patient samples, and the data were analyzed anonymously.

### PCR-based Genotyping of *cps*

PCR primers used in this study are shown in Table S1. To obtain the full sequence of NTUH-K1790N *cps*, we used 3 PCR primer pairs for *cps* conserved sequences: (1) pre-galF-F, wzc-R1 (2) CPS-1, rCPS; and (3) gnd-1F, ugd. Purified PCR products of NTUH-K1790N *cps* were sequenced using a high-throughput sequencing service by Yang-Ming Genome Research Center: High-throughput Genome Analysis Core, using the Illumina/Solexa GAII sequencing platform. *K. pneumoniae cps* gene clusters usually consist of the conserved genes located at the 5′ and 3′ ends, and the variable genes in the middle. The variable *cps* genes are usually considered to be used to distinguish different capsular types. To determine the *cps* genotype, we designed 5 sets of PCR primer pairs for *cps* genes in the variable region: *orf8*(sharing amino-acid similarity with *wzy*), *orf10*, *orf11*, *orf12*, and *wzx*, according to DNA sequencing data of NTUH-K1790N (KN2) *cps*. These primers were all listed in Table S1 and designed to amplify PCR fragments <1.5 Kb in size. The specificity of these primers was tested by PCR using 77 K-serotype reference strains (Statens Serum Institute) and KN1 as templates [20]. In brief, 3 µL of an overnight bacterial culture was added to 10 µL of water and boiled for 15 min to release DNA, followed by addition of 2 µL of 10× reaction buffer, 2.5 U *Taq* polymerase (Bioman, Taipei, Taiwan), deoxynucleoside triphosphates at final concentrations of 0.1 mM each, and primers at final concentrations of 0.4 mM each to give a final volume of 20 µL. The PCR conditions were 96°C for 3 min, followed by 30 temperature cycles of 96°C for 30 s, 53°C for 15 s, and 72°C for 30 s.

### Serotyping by Immunoblotting

Rabbit anti-KN2 antiserum was generated by a commercial company LTK BioLaboratories (Taipei, Taiwan). *K. pneumoniae* C1975 (KN2), pre-washed and resuspended in 5% formaldehyde, was used to immunize the rabbits by subcutaneous injections at Day 1, 21, 31, 41. The antiserum at Day 51 was tested for its sensitivity by Western Blot analysis. For capsular serotyping, capsules were extracted by a modified hot water-phenol extraction method [5]. In brief, 1 mL overnight-cultured bacteria was harvested and resuspended in 150 µL of water. An equal volume of hot phenol (pH 6.6) was added then vortexed vigorously. The mixture was incubated at 65°C for 20 min, followed by chloroform extraction and centrifugation. Ten µL of each capsular extract was vacuum spotted onto a nitrocellulose membrane by means of a slot blot device. The membrane was overlapped with a piece of filter and both were rinsed with Western transfer buffer containing 47.8 mM Tris, 38.6 mM glycine, 20% MeOH, and 0.037% sodium dodecyl sulfate. The membrane was dried and non-specific sites were blocked by soaking the membrane in 1× phosphate buffered saline with 0.5% Tween 20 (PBST) plus 5% milk for 1 h at room temperature. The membrane was then incubated with anti-KN2 antiserum (1∶5000 dilution) dissolved in PBST plus milk at 4°C overnight, washed 4 times with PBST for 10 min each, incubated with the secondary antibody conjugated with horseradish peroxidase (goat anti-rabbit IgG-HRP, 1∶10 000) for 1 h at room temperature, and washed 3 times with PBST for 10 min each. The ECL reagent was added for 3 min and the membrane was exposed to X-ray film in the dark.

### Phage Isolation

To isolate a phage specific for KN2 *K. pneumoniae* from the natural environment, *K. pneumoniae* Ca0507 (KN2) was co-incubated with sewage collected at Taipei City in LB broth overnight. After centrifugation, the supernatant was filtered using a 0.45 µm filter and spotted onto LB plates overlaid with *K. pneumoniae* Ca0507 (KN2) to detect phage plaques. An agar overlay method was used for isolation of a pure phage preparation and to determine phage titers [28], [30]. Single plaque isolation, elution, and re-plating were performed repeatedly.

### Determination of the Host Range of Phage/Polysaccharide Depolymerase

The spot test was performed to observe phage infection or polysaccharide depolymerase activity [31]. In brief, LB agar in a 6 or 24-well plate was overlaid with top agar that had been inoculated with 100 µL of a fresh bacterial culture. Phage or purified recombinant polysaccharide depolymerase (1 µL, 1 µg/µL) expressed by *E. coli* BL21(DE3) was spotted onto the plate after the top agar had solidified. After overnight incubation at 37°C, plates were observed for formation of lytic or semi-clear spots.

### Electron Microscopy and Pulsed Field Gel Electrophoresis (PFGE) of Phage

The purified phage were adsorbed to carbon-coated copper grids, negatively stained with 3% uranyl acetate, and observed in a Hitachi-7100 Transmission Electron Microscope (TEM) at an accelerating voltage of 100 kV. Photographs were taken at a magnification of 70,000–150,000. For PFGE, phage suspensions were mixed 1∶1 with 2% low melt agarose and poured into molding blocks. A 4-mm block slice was put into the well of a 1% agarose gel (Bio-Rad, Hercules, CA, USA), and the well was sealed with 0.8% low melt agarose. The gel was run at 14°C, 6 V/cm, 15 h, switch time 1–12 s in a Biorad CHEF MAPPER XA using 0.5× TBE buffer. The gel was stained with ethidium bromide for 10 min and washed in distilled water before visualization under UV light.

### Phage Genomic DNA Sequencing

Phage genomic DNA was extracted using a Qiagen Lamda kit (Qiagen, Valencia, CA). After phage were precipitated and lysed, the phage DNA was extracted using phenol/chloroform and then precipitated with ethanol. Genomic sequencing was performed using high-throughput sequencing by the Yang-Ming Genome Research Center: High-throughput Genome Analysis Core, using the Illumina/Solexa GAII sequencing platform. Putative open reading frames (ORFs) were predicted using the GeneMark network service (http://exon.gatech.edu/). The search for homologous genes was done using the BLAST network service (http://blast.ncbi.nlm.nih.gov/Blast.cgi?CMD=Web&PAGE_TYPE=BlastHome). The presentation of genome sequence features was done on the CGView server (http://stothard.afns.ualberta.ca/cgview_server/).

### Protein Expression and Purification

PCR fragments of the phage gene *orf96* encoding putative polysaccharide depolymerases were cloned into a pET-28c expression vector (Novagen, Madison, WI, USA) via the BamHI and SalI sites. The resulting *orf96*-pET28c plasmid was transformed into an *E. coli* BL21(DE3) strain. The recombinant His-tagged ORF96 protein was expressed under 0.05 mM IPTG induction at 16°C overnight, followed by purification from the soluble fraction using Ni-NTA (Qiagen, Valencia, CA) according to the manufacturer’s instructions and SDS-PAGE analysis.

### DNA Sequencing Data

All new data on DNA sequencing in this study has been deposited in DDBJ/EMBL/GenBank databases. The *cps* sequences of NTUH-K1790N have been deposited under the accession number AB795939. The genome sequences of phage 0507-KN2-1 have been deposited under the accession number AB797215.

## Results

### 1. Identification of *K. pneumoniae* Clinical Isolates with a Novel *cps* Genotype

**Figure 1:**
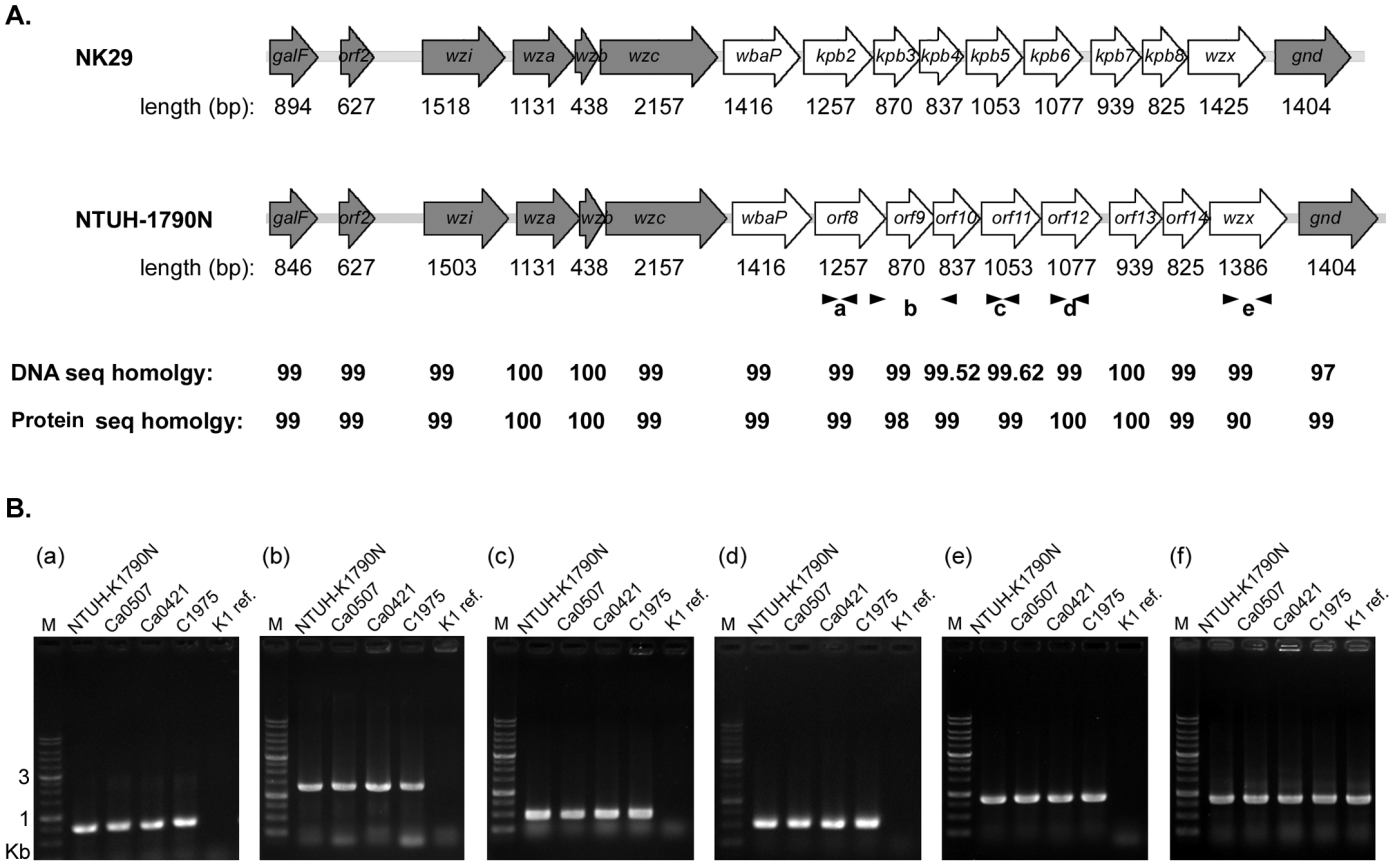
NTUH-K1790N *cps*-PCR genotyping in *K. pneumoniae*. (**A**) Comparison of the capsular polysaccharide synthesis (*cps*) regions of NK29 (Shu *et al*., 2009) and NTUH-K1790N strains. ORFs are shown by arrows, with the length as indicated below. Gray arrows show the conserved ORFs, and white arrows show the variable ORFs in different serotypes. The homology score between NK29 and NTUH-K1790N is compared using the sequence alignment program ClustalW2 (http://www.ebi.ac.uk/Tools/msa/clustalw2/). Black arrowheads refer to 5 pairs of PCR primers (a-e) used for *cps* genotyping. (**B**) *cps*-PCR genotyping of *K. pneumoniae* strains using primers as indicated: **a**. KN2-orf8-F and KN2-orf8-R; **b**. KN2-orf8-F2 and KN2-orf10-R; **c**. KN2-orf11-F and KN2-orf11-R; **d**. KN2-orf12-F and KN2-orf12-R; **e**. KN2-wzx-F and KN2-wzx-R; **f**. 23S rRNA as control for *K. pneumoniae*. K1 ref. presents the reference strain for the K1 serotype.G.

To characterize the undefined capsular type of *K. pneumoniae* NTUH-K1790N, we amplified and sequenced the *cps* region between the conserved genes *galF* and *gnd*. We found that this *cps* region of NTUH-K1790N was similar to that published for undefined strain NK29 (*cps*kpB, [32]) **(** **Figure 1A**). These 2 strains had the same *cps* gene annotation between *galF* and *gnd*, with a total DNA sequence similarity of 99%. In addition, the *cps* sequences of NTUH-K1790N and NK29 did not resemble any other publicly available *cps* sequences. To further characterize this unknown *cps* genotype, we designed PCR primers from 5 genes in the variable region of NTUH-K1790N *cps* **(** **Figure 1A**): *orf8*, *orf10*, *orf11*, *orf12*, and *wzx*. O*rf8* encoded a putative protein with 11 predicted trans-membrane domains (by SMART program, http://smart.embl-heidelberg.de/), sharing an amino-acid sequence identity of 133/409 (33%) with Wzy polymerase of *Escherichia coli* (accession ADI43271). Wzx functions as a flippase to transfer repeat units of CPS across the plasma membrane, and Wzy polymerizes these repeating units in the periplasm [33]. Because *wzx* and *wzy* have low sequence similarity, they were assumed to be serotype-specific and could thus be used for typing [32], [34]–[36]. O*rf10* and *orf12* encoded putative glycosyltransferases, which transfer sugars to specific biomolecules and are diverse in nature. O*rf11* encoded a hypothetical protein, sharing an amino-acid sequence identity of 92/342(27%) with beta-glucanase of *Bacteroides* sp. (accession ZP_07938801.1).

PCR of NTUH-K1790N *cps* was performed using these different primer pairs in 77 *Klebsiella* reference strains of known capsular serotypes from the Statens Serum Institute and in a new serotype KN1 (strain A1517) [20]. All PCR results were negative in the 77 K serotype and KN1 reference strains (data not shown), suggesting that the NTUH-K1790N *cps* gene might differ from that of the test strains. We further applied NTUH-K1790N *cps*-PCR to other clinical isolates from Asia and Canada with unknown capsular types, including isolates from UTI, blood, non-blood, and CSF. Interestingly, of the 264 clinical isolates tested, we found that 3 strains, Ca0507, Ca0421, and C1975, were positive for the NTUH-K1790N *cps* gene in all PCR analyses **(** **Figure 1B**). Ca0507 and Ca0421 were isolated from blood of septic patients in Canada [20], and C1975 was isolated from sputum of a patient in NTUH, Taiwan [7]. Hence, NTUH-K1790N *cps*-PCR actually did recognize some *K. pneumoniae* strains. Our data indicated that NTUH-K1790N, Ca0507, Ca0421, C1975, and NK29 belonged to a group of *Klebsiella* isolates with the same *cps* genotype. This *cps* genotype differs from that of the 78 previously documented types and therefore could be novel.

### 2. Characterization of the Novel Capsular Serotype of *K. pneumoniae* by Immunoblotting

**Figure 2:**
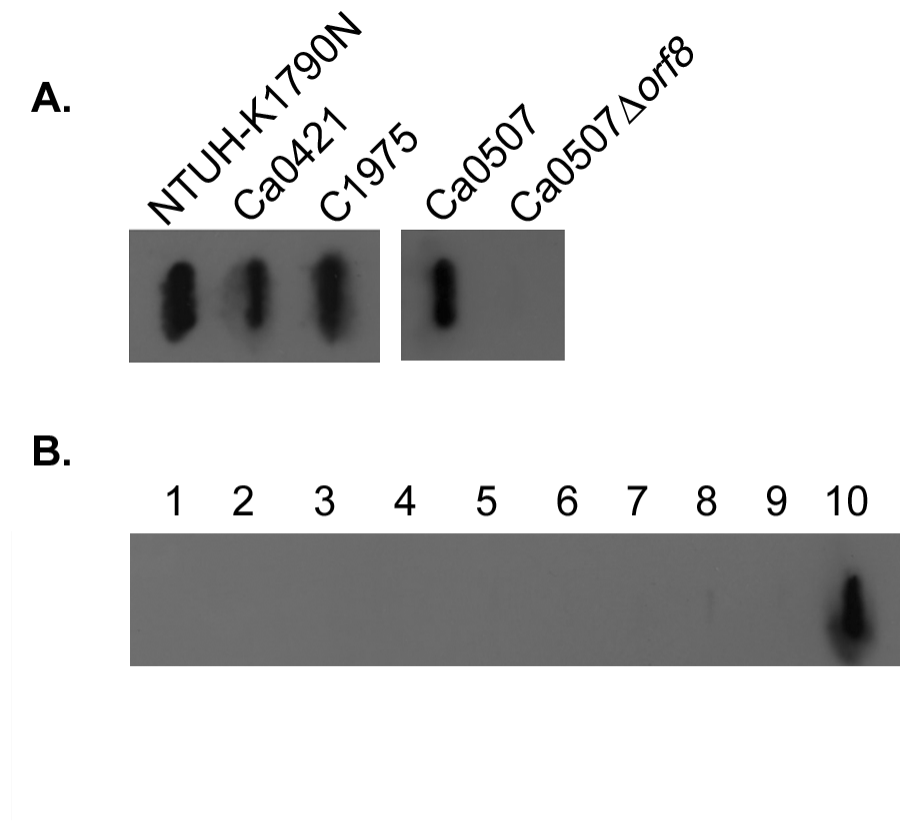
Immunoblot serotyping of *K. pneumoniae* NTUH-K1790N, Ca0507, Ca0421, and C1975. Rabbit anti-C1975 antiserum was used as the first antibody (1∶5000) and goat anti-rabbit IgG-HRP as the second antibody (1∶10 000). (**A**) The slot blot showing anti-C1975 antiserum reacts to the extracted capsular polysaccharides from strains NTUH-K1790N, C1975, Ca0421 and Ca0507. Ca0507Δ*orf8* presents the isogenic mutant with a deletion of gene *orf8* in the *cps*. (**B**) The slot blot for the extracted capsular polysaccharides from 77 K serotype reference strains and a new type A1517. Sample1–9: K31 to K39; 10: C1975 as control. Negative reactions were observed in other reference strains and A1517 (data not shown).

To further clarify whether NTUH-K1790N, Ca0507, Ca0421, C1975, and NK29 belonged to a new capsular serotype, we generated antiserum against C1975 for serotyping. Immunoblotting showed strong positive signals in the extracted capsular polysaccharides from all four strains, indicating that these four strains belonged to the same serotype (**Figure 2A** **)**. In addition, we generated an isogenic Ca0507 CPS^−^ mutant, in which *orf8* (predicted to encode Wzy) of the *cps* locus was deleted by an unmarked deletion method using the temperature-sensitive plasmid pKO3-Km [37], [38]. Immunoblot serotyping showed that this mutant was no longer recognized by anti-C1975 antiserum (**Figure 2A** **)**, confirming that *orf8* was essential for capsular polysaccharide synthesis. We thus concluded that anti-C1975 antiserum was reactive to capsular polysaccharides.

We performed immunoblot serotyping of the 77 *Klebsiella* K reference strains and A1517. The reaction to anti-C1975 antiserum was negative in all 78 strains (**Figure 2B**). These results indicate that anti-C1975 antiserum is specific and does not react to the other 78 documented capsular serotypes. This group of *K. pneumoniae* was thus determined to belong to a novel serotype. We named this new capsular serotype ‘KN2.’

### 3. Isolation of a Phage Specific for KN2 *K. pneumoniae*

**Figure 3:**
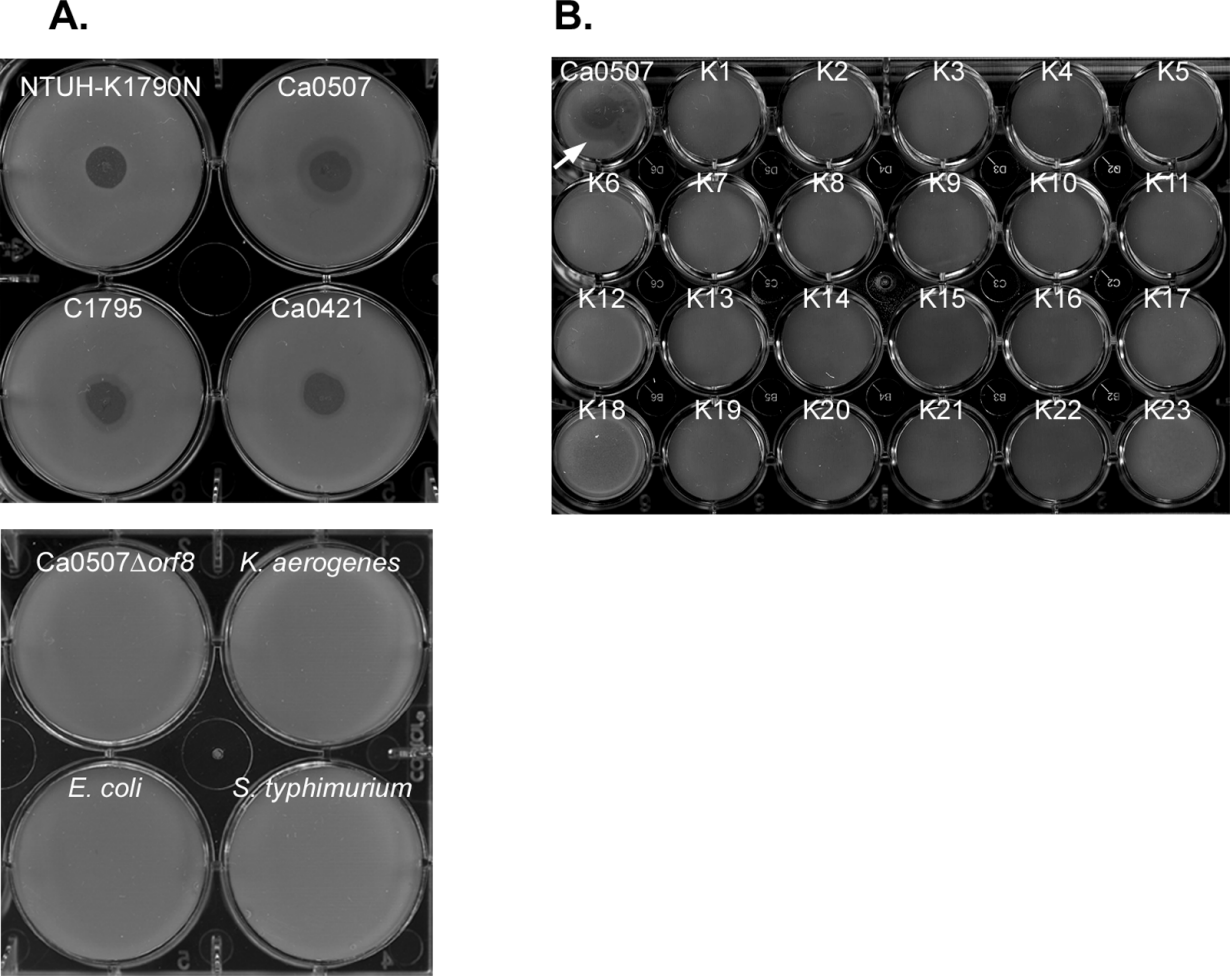
Bacterial host range of phage 0507-KN2-1. (**A**) Upper panel showing spot tests of 0507-KN2-1 (10^9^) on *K. pneumoniae* KN2 strains as indicated. Lower panel showing spot tests on Ca0507 isogenic mutant with a deletion of gene *orf8* in *cps* (Ca0507Δ*orf8*), *K. aerogenes, E. coli* 29522 and *S. typhimurium* ATCC14028. (**B**) Spot tests of 0507-KN2-1 (10^9^) on *K. pneumoniae* 77 K serotype reference strains and a new type A1517. Ca0507 was used as a positive control (indicated by a white arrow). Negative reactions were observed in all reference strains and A1517 (data on K24-K77 and A1517 not shown).

In nature, encapsulated bacteria can be infected by phages, which often carry capsule-degrading enzymes. The use of phages for *Klebsiella* capsular typing has been previously described [27], [28], [39]. Such lytic phages can also be used as therapeutic agents [40]–[43]. In this study, we sought to isolate a lytic phage of the KN2 *K. pneumoniae* strain from the natural environment. A phage was isolated from sewage collected at Taipei city by co-incubation with *K. pneumoniae* Ca0507. In spot tests, this phage caused a lytic spot on *K. pneumoniae* Ca0507 (KN2) and also on three other KN2 strains (**Figure 3A**). Lysis did not occur in the other *Enterobacteriaceae* tested, including *Klebsiella aerogenes, Escherichia coli, Salmonella typhimurium*, and interestingly, the KN2 CPS^−^ mutant. This result suggests that infection of the phage might occur via targeting and recognition of the capsular polysaccharides in *K. pneumoniae*.

When testing *K. pneumoniae* with different capsular serotypes, this bacteriophage did not cause lytic infections in any of the 77 K reference strains or in KN1 (**Figure 3B**). Therefore, this phage (named 0507-KN2-1) is specific to KN2 *K. pneumoniae* and can be used for KN2 typing. Consistent with the results of *cps*-PCR genotyping and immunoblot serotyping, phage typing indicated again that these KN2 *K. pneumoniae* strains have a novel capsular structure that differs from the 78 documented capsule types.

### 4. Morphological and Molecular Characterization of Phage 0507-KN2-1

**Figure 4:**
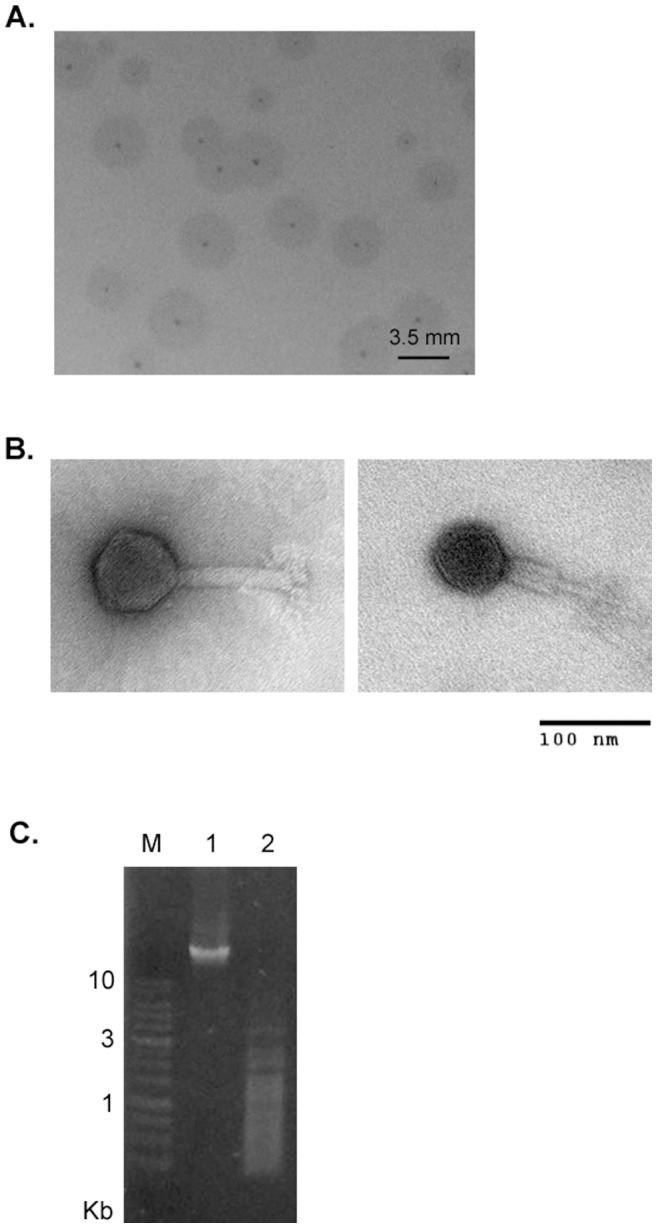
Characterization of phage 0507-KN2-1. (**A**) Plaque morphology of 0507-KN2-1 on *K. pneumoniae* Ca0507. Scale bar, 3.5 mm. (**B**) TEM images of 0507-KN2-1 taken at 150 000 × magnification. Scale bars for both pictures, 100 nm. (**C**) Restriction digestion of 0507-KN2-1 genome. M: DNA marker; lane1: uncut genomic DNA, 2 µg; lane2: genomic DNA 2 µg incubated with HinPI1 at 37°C for 5 h.

Phage 0507-KN2-1 produced plaques with small clear centers surrounded by hazy rings (halo) (**Figure 4A**), suggesting the production of soluble phage enzymes like exopolysaccharide depolymerases [44]. The morphology of purified 0507-KN2-1 phage particles were further studied using TEM (**Figure 4B**). The average length of the phage tails was approximately 107 nm, and the average diameter of the phage heads was approximately 66 nm. The morphological characteristics of 0507-KN2-1 resembled *Myoviridae* family members, which have an icosahedral head and a contractile tail [45].

We further characterized the genome of 0507-KN2-1. Analysis by PFGE showed that the phage genome has an approximate size of 145.5–194 kb (not shown). The extracted phage DNA was sensitive to digestion with the restriction enzyme HinP1I (**Figure 4C**), indicating a double-stranded DNA genome. Whole-genome sequencing of phage 0507-KN2-1 was carried out using high-throughput sequencing. We obtained a circular map of the 159 991 bp genome, which has a G+C content of 46.7% and 154 predicted ORFs encoding hypothetical proteins with at least 100 amino acid residues (Table S2). BlastN analysis revealed that the most related genome sequences in the database were *Salmonella* phage PhiSH19 (accession JN126049.1, covering 50% of the 0507-KN2-1 genome with 72–99% identity), *Salmonella* phage Vi01 (accession FQ312032.1, covering 48% of the 0507-KN2-1 genome with 72–100% identity), *Salmonella* phage SFP10 (accession HQ259103, covering 47% of the 0507-KN2-1 genome with 72–96% identity), and *Escherichia* phage vB_EcoM_CBA120 (accession JN593240.1, covering 46% of the 0507-KN2-1 genome with 72–100% identity). All of these phages are *Myoviridae* members and have genome sizes of 157–158 kb. By BlastP search, 0507-KN2-1 potential gene products showed significant sequence similarity to proteins from different phages infecting *Salmonella*, *Shigella*, *Escherichia*, and *Dickeya* (Table S2). The combined results of phage morphology, genome size, and sequence similarity suggest that 0507-KN2-1 is a *Myoviridae* virus.

### 5. Expression of a Capsular Polysaccharide Depolymerase Specific for KN2

We observed that the plaques of phage 0507-KN2-1 were surrounded by halos, indicative of bacterial cell decapsulation. This observation suggested the phage produced a depolymerase enzyme that could diffuse through the agar layer. Phage depolymerases, often a part of the tail spike or tail fiber, can degrade bacterial capsular polysaccharides into their component oligosaccharide units during infection. Capsular polysaccharide depolymerases have multiple applications, including use as therapeutic agents against bacterial pathogens [46], [47], for the prevention or eradication of biofilms [44], [48], and for the production of oligosaccharides from polysaccharides [49]–[51]. We therefore sought to isolate and purify the depolymerase of this phage.

Analysis of the genome sequence of phage 0507-KN2-1 revealed that *orf96* (3738 bp) encoded a putative protein (1245 amino acids) sharing amino-acid sequence similarity to other known phage tail fiber or spike proteins in a 170-amino-acid region of the N-terminus. For example, this coding region shares identity of 125/167 amino acids (75%) with the tail fiber protein of *Escherichia* phage PhaxI (total 927 amino acids, accession number YP_007002808.1). Interestingly, the C-terminal ∼200 amino-acid region shares a sequence similarity to the polysaccharide lyase of *Enterobacteria* phage K5 (total 632 amino acids, accession number: CAA71133.2) with an identity of 61/222 amino acids (27%).

**Figure 5:**
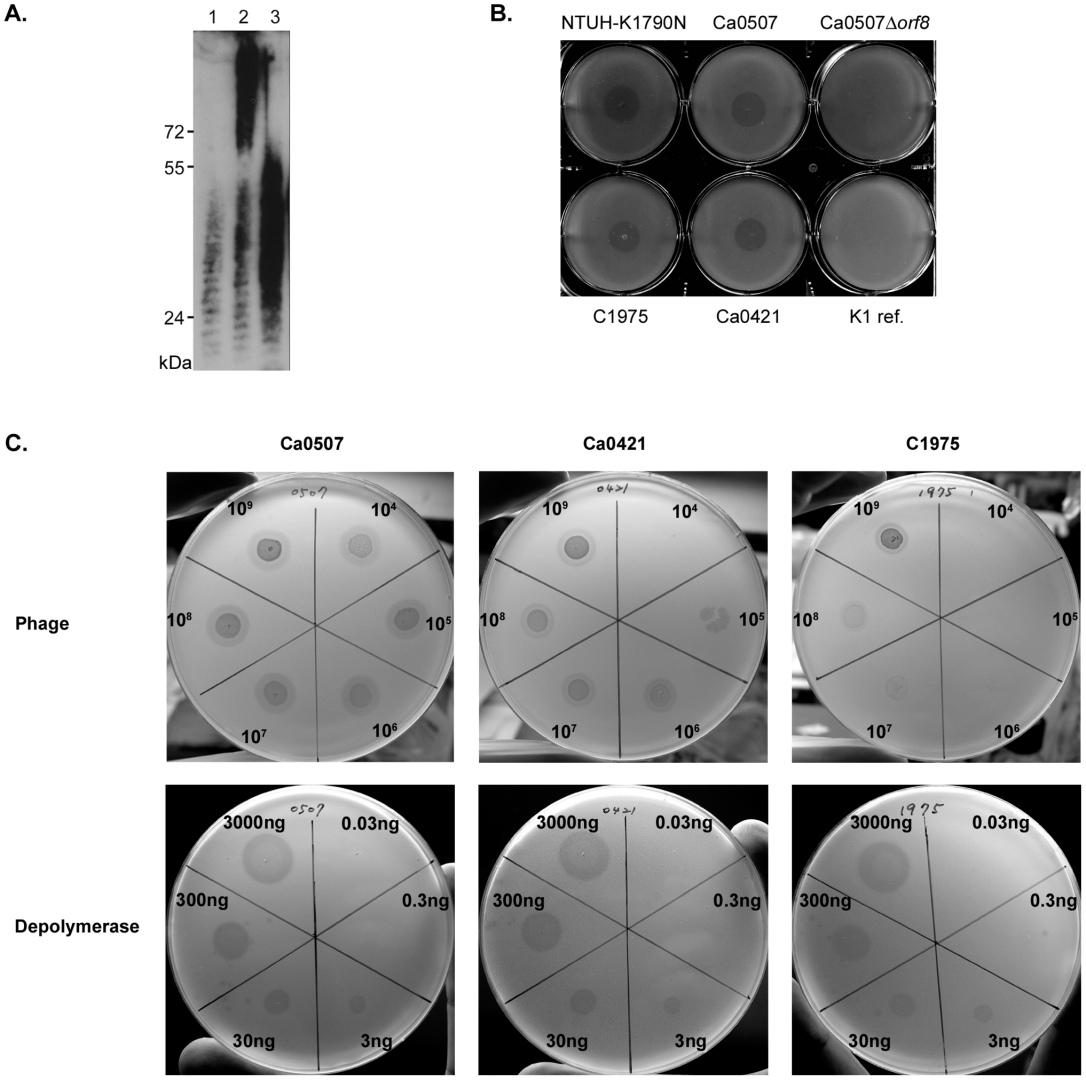
Bacteriophage-derived depolymerase specific for KN2 capsular polysaccharides. (**A**) Immunoblot showing depolymerzation of KN2 extracted capsular polysaccharides by purified ORF96 proteins. The extracted capsular polysaccharides alone or incubated with ORF96 proteins (10 µg) at 37°C for 6 h were separated by SDS-PAGE, transferred onto a nitrocellulose membrane and detected using rabbit anti-C1975 antiserum (1∶5000) and goat anti-rabbit IgG-HRP (1∶10 000). Lane1: Ca0507Δ*orf8* alone; lane2: wild-type Ca0507 alone; lane3: wild-type Ca0507 plus ORF96 proteins. (**B**) A spot test showing ORF96 proteins with the depolymerase activity to KN2. Purified ORF96 proteins (1 µg) was spotted on the bacterial lawn and incubated at 37°C overnight. Decapsulation of KN2 *K. pneumoniae* NTUH-K1790N, Ca0507, Ca0421 and C1975 was observed. K1 ref. presents the reference strain of the K1 serotype. (**C**) Comparison of sensitivity of phage 0507-KN2-1 and capsular polysaccharide depolymerase. Spot tests with serial titration (counter-clockwise) of phage 0507-KN2-1 (upper panels) or purified ORF96 proteins (lower panels) on KN2 strains as indicated.

To determine whether the product of this gene had depolymerase activity, we cloned *orf96* into the pET28c expression vector. His-tagged ORF96 protein with a predicted size of ∼138 kDa was expressed and purified. Immunoblotting showed that the purified protein caused depolymerization of isolated KN2 capsular polysaccharides (**Figure 5A**). In spot tests, purified ORF96 protein caused decapsulation in the KN2 *K. pneumoniae* strains (**Figure 5B**) but in none of the 77 K reference strains or KN1 (data not shown). In addition, the purified ORF96 protein did not cause digestion-like spots on the KN2 CPS^−^ mutant (Ca0507Δ*orf8*) bacterial lawns. Therefore, this depolymerase was specific for KN2 capsular polysaccharides. In contrast to phage infection, the purified depolymerase exhibited a consistent sensitivity between different KN2 strains (**Figure 5C**), suggesting that the depolymerase was more ideal for typing than phage did. After validation of the sensitivity and specificity in more strains, this KN2 polysaccharide depolymerase could be considered its application to large-scale capsular typing for *K. pneumoniae* clinical strains.

## Discussion

The capsule, one of the most important *K. pneumoniae* virulence factors, protects the bacterium from the lethal serum factors and phagocytosis that are part of the host immune response. Based on the structural variability of the capsular polysaccharides, *Klebsiella* sp. has been traditionally classified into 77 K serotypes for several decades [52]. No further capsular serotypes were reported until recently, when a new serotype (KN1) was found in our lab during studies of PLA-inducing *K. pneumoniae* [20]. Through serological and molecular typing, the pathogenicity and epidemiological relevance of the different capsular serotypes of *K. pneumoniae* in various infectious diseases have been identified [53]–[56]. In this study, we found a UTI strain with a capsular type that was initially not recognized using immunoserotyping or *cps*-PCR. Our findings showed that this strain had a novel capsular type, which we called KN2. Our experience suggests that the inability to type some clinical strains may be due to the presence of new capsular types that have not yet been identified and that more *Klebsiella* capsule types may exist than are presently known.

Our data indicated that *K. pneumoniae* KN2 strains had the same *cps* genotype and shared capsule structural characteristics, including reactivity to antiserum, susceptibility to phage infection, and decapsulation by a capsular polysaccharide depolymerase. These features distinguish the KN2 strains from the documented K reference strains. No cross-reactions were observed between KN2 strains and the other 79 documented capsular types in any our assays, indicating the novelty of their capsule structures and a high degree of specificity in typing. The prevalence of this new capsular type in 265 clinical isolates of *K. pneumoniae* from Asia and Canada is ∼1.5%, including 1 isolate from UTI, 2 bacteremic isolates, and 1 non-blood isolate.

Serological diagnosis is often used to identify *Klebsiella* capsular serotypes, but this method has drawbacks. The antisera are usually expensive, and their limited sensitivity and specificity result in inconsistent results in serotype prevalence studies [21], [22], [57], [58]. Molecular typing is more accurate but requires type-specific sequence data for the *cps* regions, which are not yet available for all types up to now. Phage-based capsular typing is rapid, simple, and low-cost, but requires phage with a narrow host range and no cross-reactivity to other types. Here, we further isolated and purified a phage enzyme that can be used for efficient typing. The stability and sensitivity of purified KN2 depolymerase for inducing decapsulation spots is better than phage infection, and the amount of enzyme used can be easily quantified. Therefore, enzyme-based typing is more suitable for large-scale epidemiological studies of capsular type prevalence. Using this more efficient typing method, the prevalence of KN2 capsular type in different *K. pneumoniae*-mediated diseases can be further investigated.

Phage 0507-KN2-1 is a double-stranded DNA virus that belongs to *Myoviridae.* This virus shares genome characteristics with other phages that infect *Enterobacteriaceae*, such as *Salmonella* phage Vi01-related viruses. To date, all *K. pneumoniae* phages with published whole genome sequences in the database belonged to 3 families in the tailed dsDNA *Caudovirales* order: *Podoviridae*, *Siphoviridae,* and *Myoviridae.* Phage 0507-KN2-1 shares less genome similarity with previously reported *K. pneumoniae* phages KP34 (*Podoviridae*, 43.81 kb, accession GQ413938), phiKO2 (*Siphoviridae*, 51.6 kb, accession AY374448), KP15 (*Myoviridae*, 174.44 kb, accession GU295964), K11 (*Podoviridae*, 41.18 kb, accession EU734173), KP32 (*Podoviridae*, 41.12 kb, accession GQ413937), KP36 (*Siphoviridae*, 49.82 kb, accession JQ267364), and JD001 (*Myoviridae*, 48.81 kb, accession JX866719). Although several *K. pneumoniae* phages have been reported, associations between the molecular characteristics of the phage or polysaccharide depolymerase and the specificity to host capsular serotypes have not been clearly described.

In this study, we provide molecular data for the phage and polysaccharide depolymerase together with well-defined characteristics and host specificity. The host range of phage 0507-KN2-1 is narrow and specific, only to *K. pneumoniae* that have the KN2 capsular type. In the first step of infection, phages need to attach and absorb to specific targets on the bacterial surface. Our results indicate that capsular polysaccharides may be the specific receptors for 0507-KN2-1, since neither the phage nor the depolymerase causes lytic or semi-turbid spots on Ca0507Δ*orf8* mutant. The specificity of 0507-KN2-1 could be due to the phage-carried polysaccharide depolymerase, which can recognize the KN2 capsular structure distinct from other capsular types. The well-characterized host specificity and full sequences of phage and polysaccharide depolymerase revealed by this study will be helpful for elucidating the mechanistic details that determine the host range of this virus. In addition, KN2 depolymerase, for which we have provided the sequence data and demonstrated *in vitro* activity, can be used to investigate its functional regions, the structure of KN2 capsule polysaccharides, and the interactions between this enzyme and its polysaccharide substrates.

In many hospitals, *K. pneumoniae* is the second most frequent etiological agent of Gram-negative bacteraemia and UTIs. Drug resistant isolates remain a problem, rendering current antibiotic therapy relatively ineffective. *K. pneumoniae* reportedly produces extended-spectrum β-lactamases, resulting in multidrug resistance [59]–[62]. In addition, development of antibiotic resistance can be enhanced by formation of protective biofilms that can impede the penetration of drugs. Thus, alternative therapeutic methods are needed. Lytic phages have been considered possible alternatives to antibiotics for treating *Klebsiella* infections [40], [41], [63]. The narrow host range displayed by phages can be advantageous for use as therapeutic agents since it has limited adverse effects on the natural bacterial population compared to chemical antibiotics. Administration of capsular depolymerase has been shown to reduce bacteraemia and deaths in animals [46] and to exhibit protective effects by promoting *in vivo* killing of bacteria by neutrophils [47]. We found KN2 possessed by clinical *K. pneumoniae* causing bacteraemia and UTIs. The potential therapeutic effects of phage 0507-KN2-1 and recombinant KN2 depolymerase can be further investigated for use in so-called phage therapy and glycosidase therapy. Because the mechanism of bacterial killing by lytic phage and glycosidases differ from that of antibiotics, these agents have the potential to treat antibiotic-resistant *K. pneumoniae* strains. Moreover, their ability to generate oligosaccharides from polysaccharides could be applied to polysaccharide vaccine production.

In summary, we have identified and characterized a new capsular type of *K. pneumoniae*, its specific bacteriophage, and the polysaccharide depolymerase of this phage. These results may be applicable to capsular typing in further epidemiological investigations and to the development of new treatments for antibiotic-resistant *K. pneumoniae* infections.

## Supporting Information

Table S1 **PCR Primers used in this study.**
(DOC)

Table S2 **Annotation and features of predicted ORFs in bacteriophage Ca0507-KN2 genome.**
(DOC)
