# Supplementary material for: Isolation of a Bacteriophage Specific for a New Capsular Type of Klebsiella pneumoniae and Characterization of Its Polysaccharide Depolymerase
Source: PLoS One. 2013 Aug 2;8(8):e70092. doi: 10.1371/journal.pone.0070092 (PMC3732264; doi:10.1371/journal.pone.0070092)
Supplement: Table S1 — PCR Primers used in this study. (DOC) [file pone.0070092.s001.doc]

**Table S1. PCR Primers used in this study.**

| Primer name | Sequences | Position | Purpose | Fragment size (bp) | Source or references |
| --- | --- | --- | --- | --- | --- |
| pre-galF-F | GAGCCGCTGAATAACCTGAA | upstream of *galF* | *cps* 5’PCR | ~8000 | [20] |
| wzc-R1 | GCTTCCATCATTGCAAAATG | *wzc* | *cps* 5’PCR |  | this study |
| CPS-1 | GCTGGTAGCTGTTAAGCCAGGGGCGGTAGCG | upstream of *wzi* | *cps* middle fragment PCR | ~17000 | [20] |
| rCPS | TATTCATCAGAAGCAGCACGCAGCTGGGAGAAG CC | *gnd* | *cps* middle fragment PCR |  | [20] |
| gnd-1F | GTTGTCGGTATGGCTGTGATGG | *gnd* | *cps* 3’PCR | ~2000 | [20] |
| ugd | CGCGTTCGGGTTGATCTCTG | *ugd* | *cps* 3’PCR |  | [20] |
| KN2-orf8-F | CACTACCTATGCTGATGCTATAAGTG | *wzy* of 1790N | *cps*-PCR genotyping for KN2 | 726 | this study |
| KN2-orf8-R | GCATTAGCAGAAATCGATGTCAAAGAG | *wzy* of 1790N | *cps*-PCR genotyping for KN2 |  | this study |
| KN2-orf8-F2 | CCTATTAGGAACAATGTGGACCGG | *wzy* of 1790N | *cps*-PCR genotyping for KN2 | 1245 | this study |
| KN2-orf10-R | CATTTAGAGCACGTCCTAGTCCCAC | *orf10* of 1790N | cps-PCR genotyping for KN2 |  | this study |
| KN2-orf11-F | CTCGCATAGTGGCTCGATAA | *orf11* of 1790N | cps-PCR genotyping for KN2 | 468 | this study |
| KN2-orf11-R | TCCCGCAGTTCTTGGCCTAG | *orf11* of 1790N | cps-PCR genotyping for KN2 |  | this study |
| KN2-orf12-F | GTTCCCACGATTGATATAGTACTTC | *orf12* of 1790N | *cps*-PCR genotyping for KN2 | 506 | this study |
| KN2-orf12-R | CTACGTATGTGTTCAGGTAAG | *orf12* of 1790N | *cps*-PCR genotyping for KN2 |  | this study |
| KN2-wzx-F | CAATTGCAAGTATTACAAGTACGC | *wzx* of 1790N | *cps*-PCR genotyping for KN2 | 850 | this study |
| KN2-wzx-R | TTACCACCCAACAGCCTCATAGCG | *wzx* of 1790N | *cps*-PCR genotyping for KN2 |  | this study |
