# Supplementary material for: Isolation of a Bacteriophage Specific for a New Capsular Type of Klebsiella pneumoniae and Characterization of Its Polysaccharide Depolymerase
Source: PLoS One. 2013 Aug 2;8(8):e70092. doi: 10.1371/journal.pone.0070092 (PMC3732264; doi:10.1371/journal.pone.0070092)
Supplement: Table S2 — Annotation and features of predicted ORFs in bacteriophage Ca0507-KN2 genome. (DOC) [file pone.0070092.s002.doc]

**Table S2. Annotation and features of predicted ORFs in bacteriophage Ca0507-KN2 genome.**

| ORF*^a^* | Strand*^b^* | Position | | Size (aa) | ORF homolog characteristic(s) | E-value | Sequence identity*^c^*(no. of matching aa/total no. of aa) | GenBank accession no. |
| --- | --- | --- | --- | --- | --- | --- | --- | --- |
|  |  | Start | Stop |  |  |  |  |  |
| 1 | + | 1 | 2328 | 775 | Gp46 recombination endonuclease subunit [*Shigella* phage phiSboM-AG3] | 0.0 | 526/774 (68%) | [YP_003358594.1](http://www.ncbi.nlm.nih.gov/protein/282599282?report=genbank&log$=prottop&blast_rank=1&RID=2ZTRK3SD016) |
| 2 | + | 2483 | 2803 | 106 | NrdA.1 conserved hypothetical protein [*Shigella* phage phiSboM-AG3] | 2e-61 | 92/104 (88%) | [YP_003358592.1](http://www.ncbi.nlm.nih.gov/protein/282599280?report=genbank&log$=prottop&blast_rank=1&RID=2ZTRK3SD016) |
| 3 | + | 3172 | 3789 | 205 | conserved uncharacterised protein [*Salmonella* phage Vi01] | 2e-128 | 171/205 (83%) | [YP_004327468.1](http://www.ncbi.nlm.nih.gov/protein/326804597?report=genbank&log$=prottop&blast_rank=1&RID=2ZTRK3SD016) |
| 4 | + | 3789 | 4427 | 212 | orf113 gene product [*Escherichia* phage vB_EcoM_CBA120] | 2e-127 | 176/212(83%) | [YP_004957791.1](http://www.ncbi.nlm.nih.gov/protein/372199455?report=genbank&log$=prottop&blast_rank=1&RID=EZV2PS44015) |
| 5 | + | 4824 | 5171 | 115 | hypothetical protein [*Shigella* phage phiSboM-AG3] | 4e-35 | 54/97 (56%) | [YP_003358586.1](http://www.ncbi.nlm.nih.gov/protein/282599274?report=genbank&log$=prottop&blast_rank=1&RID=2ZRM677K01S) |
| 6 | + | 5248 | 7608 | 786 | phage associated protein [*Salmonella* phage Vi01] | 0.0 | 502/814 (62%) | [YP_004327465.1](http://www.ncbi.nlm.nih.gov/protein/326804594?report=genbank&log$=prottop&blast_rank=1&RID=2ZRM677K01S) |
| 7 | + | 7636 | 8229 | 197 | hypothetical protein [*Shigella* phage phiSboM-AG3] | 3e-115 | 160/188 (85%) | [YP_003358582.1](http://www.ncbi.nlm.nih.gov/protein/282599270?report=genbank&log$=prottop&blast_rank=1&RID=2ZRM677K01S) |
| 8 | + | 8533 | 8886 | 117 | MobD.6 conserved hypothetical phage protein [*Shigella* phage phiSboM-AG3] | 4e-70 | 100/117(85%) | [YP_003358580.1](http://www.ncbi.nlm.nih.gov/protein/282599268?report=genbank&log$=prottop&blast_rank=2&RID=FAA2TAVN014) |
| 9 | + | 8948 | 9556 | 202 | hypothetical protein [*Salmonella* phage PhiSH19] | 1e-105 | 147/206(71%) | [YP_007008051.1](http://www.ncbi.nlm.nih.gov/protein/422937849?report=genbank&log$=prottop&blast_rank=1&RID=FAA2TAVN014) |
| 10 | + | 9556 | 10620 | 354 | Gp61 DNA primase subunit [*Salmonella* phage Vi01] | 0.0 | 299/354(84%) | [YP_004327461.1](http://www.ncbi.nlm.nih.gov/protein/326804590?report=genbank&log$=prottop&blast_rank=1&RID=FAA2TAVN014) |
| 11 | + | 11013 | 11501 | 162 | putative uncharacterised protein [*Salmonella* phage Vi01] | 4e-72 | 114/162(70%) | [YP_004327460.1](http://www.ncbi.nlm.nih.gov/protein/326804589?report=genbank&log$=prottop&blast_rank=2&RID=FW9DDNFZ013) |
| 12 | + | 11895 | 12689 | 264 | phage-encoded peptidoglycan binding protein [*Salmonella* phage Vi01] | 6e-166 | 223/263(85%) | [YP_004327457.1](http://www.ncbi.nlm.nih.gov/protein/326804586?report=genbank&log$=prottop&blast_rank=2&RID=FW9DDNFZ013) |
| 13 | + | 12796 | 13638 | 280 | PhoH-like protein [*Shigella* phage phiSboM-AG3] | 3e-180 | 241/280(86%) | [YP_003358571.1](http://www.ncbi.nlm.nih.gov/protein/282599259?report=genbank&log$=prottop&blast_rank=1&RID=FW9DDNFZ013) |
| 14 | + | 13705 | 14364 | 219 | putative homing endonuclease [*Shigella* phage phiSboM-AG3] | 5e-73 | 120/231(52%) | [YP_003358572.1](http://www.ncbi.nlm.nih.gov/protein/282599260?report=genbank&log$=prottop&blast_rank=1&RID=FW9DDNFZ013) |
| 15 | + | 14426 | 16711 | 761 | ribonucleotide-diphosphate reductase alpha subunit [*Escherichia* phage PhaxI] | 0.0 | 660/758(87%) | [YP_007002722.1](http://www.ncbi.nlm.nih.gov/protein/418488712?report=genbank&log$=prottop&blast_rank=4&RID=FWCV7XG3013) |
| 16 | + | 16786 | 17889 | 367 | ribonucleotide reductase of class Ia (aerobic) beta subunit [*Salmonella* phage SKML-39] | 0.0 | 333/367(91%) | [YP_007236187.1](http://www.ncbi.nlm.nih.gov/protein/431809291?report=genbank&log$=prottop&blast_rank=1&RID=FWCV7XG3013) |
| 17 | + | 18217 | 18672 | 151 | unnamed protein product [*Salmonella* phage SFP10] | 4e-72 | 105/152(69%) | [YP_004895245.1](http://www.ncbi.nlm.nih.gov/protein/351518517?report=genbank&log$=prottop&blast_rank=1&RID=FWCV7XG3013) |
| 18 | + | 18679 | 19047 | 122 | hypothetical protein [*Dickeya* phage vB_DsoM_LIMEstone1] | 1e-06 | 20/56(36%) | [CCD57624.1](http://www.ncbi.nlm.nih.gov/protein/384228660?report=genbank&log$=prottop&blast_rank=1&RID=FWCV7XG3013) |
| 19 | - | 19030 | 19410 | 126 | putative Gp25 baseplate wedge subunit [*Shigella* phage phiSboM-AG3] | 2e-70 | 104/126(83%) | [YP_003358564.1](http://www.ncbi.nlm.nih.gov/protein/282599252?report=genbank&log$=prottop&blast_rank=2&RID=FWCV7XG3013) |
| 20 | - | 19474 | 21084 | 536 | Gp5 baseplate hub subunit and tail lysozyme [*Shigella* phage phiSboM-AG3] | 0.0 | 457/536(85%) | [YP_003358563.1](http://www.ncbi.nlm.nih.gov/protein/282599251?report=genbank&log$=prottop&blast_rank=2&RID=FWCV7XG3013) |
| 21 | - | 21590 | 22393 | 267 | baseplate hub subunit [*Salmonella* phage PhiSH19] | 7e-141 | 189/267(71%) | [YP_007008040.1](http://www.ncbi.nlm.nih.gov/protein/422937838?report=genbank&log$=prottop&blast_rank=1&RID=FWCV7XG3013) |
| 22 | + | 22443 | 22973 | 176 | orf089 gene product [*Escherichia* phage vB_EcoM_CBA120] | 2e-89 | 121/174(70%) | [YP_004957771.1](http://www.ncbi.nlm.nih.gov/protein/372199435?report=genbank&log$=prottop&blast_rank=1&RID=FWCV7XG3013) |
| 23 | + | 22945 | 23433 | 162 | putative uncharacterized protein [*Salmonella* phage PhiSH19] | 1e-70 | 99/162(61%) | [YP_007008038.1](http://www.ncbi.nlm.nih.gov/protein/422937836?report=genbank&log$=prottop&blast_rank=1&RID=FWCV7XG3013) |
| 24 | + | 23472 | 24083 | 203 | putative holliday junction resolvase RuvC [*Dickeya* phage vB_DsoM_LIMEstone1] | 1e-115 | 159/200(80%) | [CCD57617.1](http://www.ncbi.nlm.nih.gov/protein/384228653?report=genbank&log$=prottop&blast_rank=3&RID=FWA7RTPK013) |
| 25 | + | 25185 | 26231 | 348 | single stranded DNA-binding protein [*Escherichia* phage ECML-4] | 0.0 | 264/350(75%) | [AFO10258.1](http://www.ncbi.nlm.nih.gov/protein/397133749?report=genbank&log$=prottop&blast_rank=1&RID=FWA7RTPK013) |
| 26 | + | 26274 | 26678 | 134 | n/a |  |  |  |
| 27 | - | 26701 | 27708 | 335 | baseplate tail tube [*Salmonella* phage PhiSH19] | 0.0 | 292/329(89%) | [YP_007008033.1](http://www.ncbi.nlm.nih.gov/protein/422937831?report=genbank&log$=prottop&blast_rank=1&RID=FWA7RTPK013) |
| 28 | + | 27702 | 28409 | 235 | Gp2 DNA end protector protein [*Salmonella* phage Vi01] | 4e-160 | 216/232(93%) | [YP_004327436.1](http://www.ncbi.nlm.nih.gov/protein/326804565?report=genbank&log$=prottop&blast_rank=1&RID=FWA7RTPK013) |
| 29 | + | 28485 | 29219 | 244 | putative uncharacterised protein [*Salmonella* phage Vi01] | 3e-31 | 88/251(35%) | [YP_004327435.1](http://www.ncbi.nlm.nih.gov/protein/326804564?report=genbank&log$=prottop&blast_rank=1&RID=FWCX784A01R) |
| 30 | + | 29242 | 29553 | 103 | hypothetical protein [*Salmonella* phage SKML-39] | 2e-37 | 56/92(61%) | [YP_007236207.1](http://www.ncbi.nlm.nih.gov/protein/431809311?report=genbank&log$=prottop&blast_rank=1&RID=FWCX784A01R) |
| 31 | + | 29691 | 30602 | 303 | unnamed protein product [*Salmonella* phage SFP10] | 4e-144 | 215/309(70%) | [YP_004895228.1](http://www.ncbi.nlm.nih.gov/protein/351518500?report=genbank&log$=prottop&blast_rank=1&RID=FWCX784A01R) |
| 32 | + | 30677 | 31339 | 220 | hypothetical protein [*Escherichia* phage PhaxI] | 5e-131 | 174/219(79%) | [YP_007002699.1](http://www.ncbi.nlm.nih.gov/protein/418488690?report=genbank&log$=prottop&blast_rank=1&RID=FWCX784A01R) |
| 33 | + | 31339 | 32394 | 351 | putative thymidylate synthase [*Shigella* phage phiSboM-AG3] | 0.0 | 269/349(77%) | [YP_003358546.1](http://www.ncbi.nlm.nih.gov/protein/282599234?report=genbank&log$=prottop&blast_rank=1&RID=FWCX784A01R) |
| 34 | + | 32391 | 32957 | 188 | putative uncharacterised protein [*Salmonella* phage Vi01] | 8e-99 | 142/190(75%) | [YP_004327430.1](http://www.ncbi.nlm.nih.gov/protein/326804559?report=genbank&log$=prottop&blast_rank=1&RID=FWCX784A01R) |
| 35 | + | 32954 | 33508 | 184 | putative dUTP diphosphatase [*Dickeya* phage vB_DsoM_LIMEstone1] | 1e-128 | 175/184(95%) | [CCD57604.1](http://www.ncbi.nlm.nih.gov/protein/384228640?report=genbank&log$=prottop&blast_rank=1&RID=FWCX784A01R) |
| 36 | + | 33508 | 34026 | 172 | putative uncharacterized protein [*Salmonella* phage PhiSH19] | 2e-36 | 75/176(43%) | [YP_007008024.1](http://www.ncbi.nlm.nih.gov/protein/422937822?report=genbank&log$=prottop&blast_rank=1&RID=FWCX784A01R) |
| 37 | + | 34011 | 35102 | 363 | UvsX RecA-like recombination protein [*Salmonella* phage Vi01] | 0.0 | 300/363(83%) | [YP_004327427.1](http://www.ncbi.nlm.nih.gov/protein/326804556?report=genbank&log$=prottop&blast_rank=1&RID=FWCX784A01R) |
| 38 | + | 35077 | 35409 | 110 | unnamed protein product [*Salmonella* phage SFP10] | 3e-44 | 74/108(69%) | [YP_004895221.1](http://www.ncbi.nlm.nih.gov/protein/351518493?report=genbank&log$=prottop&blast_rank=1&RID=FWCX784A01R) |
| 39 | + | 35416 | 36843 | 475 | DNA primase-helicase subunit [*Escherichia* phage PhaxI] | 0.0 | 428/475(90%) | [YP_004895220.1](http://www.ncbi.nlm.nih.gov/protein/351518492?report=genbank&log$=prottop&blast_rank=1&RID=FWHVVM98013) |
| 40 | + | 36906 | 37241 | 111 | hypothetical protein [*Shigella* phage phiSboM-AG3] | 8e-41 | 70/111(63%) | [YP_003358539.1](http://www.ncbi.nlm.nih.gov/protein/282599227?report=genbank&log$=prottop&blast_rank=1&RID=FWHVVM98013) |
| 41 | + | 37255 | 37569 | 104 | unnamed protein product [*Salmonella* phage SFP10] | 2e-52 | 76/97(78%) | [YP_004895218.1](http://www.ncbi.nlm.nih.gov/protein/351518490?report=genbank&log$=prottop&blast_rank=1&RID=FWHVVM98013) |
| 42 | + | 37688 | 38881 | 397 | conserved uncharacterised protein [*Salmonella* phage Vi01] | 0.0 | 271/400(68%) | [YP_004327422.1](http://www.ncbi.nlm.nih.gov/protein/326804551?report=genbank&log$=prottop&blast_rank=2&RID=FYT1PX2M016) |
| 43 | + | 39691 | 41112 | 473 | DNA ligase [*Escherichia* phage PhaxI] | 0.0 | 395/474(83%) | [YP_007002682.1](http://www.ncbi.nlm.nih.gov/protein/418488673?report=genbank&log$=prottop&blast_rank=2&RID=FYT1PX2M016) |
| 44 | + | 41587 | 42249 | 220 | Gp59 Loader of T4-like helicase [*Salmonella* phage Vi01] | 1e-130 | 171/220(78%) | [YP_004327409.1](http://www.ncbi.nlm.nih.gov/protein/326804538?report=genbank&log$=prottop&blast_rank=1&RID=FYNSP8ZJ01R) |
| 45 | - | 42252 | 44237 | 661 | putative tail length tape measure protein [*Dickeya* phage vB_DsoM_LIMEstone1] | 0.0 | 437/665(66%) | [CCD57588.1](http://www.ncbi.nlm.nih.gov/protein/384228624?report=genbank&log$=prottop&blast_rank=1&RID=FYNSP8ZJ01R) |
| 46 | - | 44248 | 45636 | 462 | hypothetical protein [*Salmonella* phage PhiSH19] | 0.0 | 403/462(87%) | [YP_007008012.1](http://www.ncbi.nlm.nih.gov/protein/422937810?report=genbank&log$=prottop&blast_rank=1&RID=FYNSP8ZJ01R) |
| 47 | - | 45633 | 46190 | 185 | baseplate wedge subunit [*Salmonella* phage PhiSH19] | 6e-114 | 151/185(82%) | [YP_007008011.1](http://www.ncbi.nlm.nih.gov/protein/422937809?report=genbank&log$=prottop&blast_rank=2&RID=FYNSP8ZJ01R) |
| 48 | - | 46205 | 47173 | 322 | Gp48 T4-like baseplate tail tube cap [*Salmonella* phage Vi01] | 0.0 | 312/322(97%) | [YP_004327405.1](http://www.ncbi.nlm.nih.gov/protein/326804534?report=genbank&log$=prottop&blast_rank=1&RID=FYNSP8ZJ01R) |
| 49 | + | 47227 | 47844 | 205 | Gp4 head completion protein [*Salmonella* phage Vi01] | 8e-124 | 173/206(84%) | [YP_004327404.1](http://www.ncbi.nlm.nih.gov/protein/326804533?report=genbank&log$=prottop&blast_rank=2&RID=FYNSP8ZJ01R) |
| 50 | - | 48052 | 48441 | 129 | hypothetical membrane protein [*Shigella* phage phiSboM-AG3] | 5e-67 | 102/129(79%) | [YP_003358515.1](http://www.ncbi.nlm.nih.gov/protein/282599203?report=genbank&log$=prottop&blast_rank=2&RID=FYNSP8ZJ01R) |
| 51 | - | 48470 | 48976 | 168 | Cd allosteric enzyme [*Shigella* phage phiSboM-AG3] | 1e-86 | 125/167(75%) | [YP_003358514.1](http://www.ncbi.nlm.nih.gov/protein/282599202?report=genbank&log$=prottop&blast_rank=2&RID=FYNSP8ZJ01R) |
| 52 | - | 48969 | 49313 | 114 | hypothetical protein [*Salmonella* phage SKML-39] | 4e-26 | 50/112(45%) | [YP_007236240.1](http://www.ncbi.nlm.nih.gov/protein/431809344?report=genbank&log$=prottop&blast_rank=1&RID=FYNSP8ZJ01R) |
| 53 | - | 49376 | 49771 | 131 | orf035 gene product [*Escherichia* phage vB_EcoM_CBA120] | 2e-20 | 53/116(46%) | [YP_004957731.1](http://www.ncbi.nlm.nih.gov/protein/372199395?report=genbank&log$=prottop&blast_rank=1&RID=FYNSP8ZJ01R) |
| 54 | - | 49768 | 50139 | 123 | hypothetical protein [*Shigella* phage phiSboM-AG3] | 3e-53 | 81/123(66%) | [YP_003358511.1](http://www.ncbi.nlm.nih.gov/protein/282599199?report=genbank&log$=prottop&blast_rank=1&RID=G1F4K92R01R) |
| 55 | - | 50136 | 50531 | 131 | n/a |  |  |  |
| 56 | - | 50528 | 51100 | 190 | putative serine/threonine protein phosphatase [*Shigella* phage phiSboM-AG3] | 4e-63 | 101/191(53%) | [YP_003358510.1](http://www.ncbi.nlm.nih.gov/protein/282599198?report=genbank&log$=prottop&blast_rank=2&RID=G1F4K92R01R) |
| 57 | - | 51145 | 51813 | 222 | putative uncharacterised protein [*Salmonella* phage Vi01] | 1e-84 | 123/221(56% | [YP_004327394.1](http://www.ncbi.nlm.nih.gov/protein/326804523?report=genbank&log$=prottop&blast_rank=2&RID=G1F4K92R01R) |
| 58 | - | 52397 | 53026 | 209 | DexA exonuclease [*Shigella* phage phiSboM-AG3] | 4e-137 | 188/208(90%) | [YP_003358507.1](http://www.ncbi.nlm.nih.gov/protein/282599195?report=genbank&log$=prottop&blast_rank=4&RID=G1Y9WV0X013) |
| 59 | - | 53019 | 53624 | 201 | Tk.4 conserved hypothetical phage protein [*Escherichia* phage vB_EcoM_CBA120] | 3e-99 | 138/190(73%) | [YP_004957722.1](http://www.ncbi.nlm.nih.gov/protein/372199386?report=genbank&log$=prottop&blast_rank=3&RID=G1Y9WV0X013) |
| 60 | - | 54134 | 54538 | 134 | Arn.3 conserved hypothetical protein [*Shigella* phage phiSboM-AG3] | 2e-73 | 110/136(81%) | [YP_003358504.1](http://www.ncbi.nlm.nih.gov/protein/282599192?report=genbank&log$=prottop&blast_rank=1&RID=G1Y9WV0X013) |
| 61 | - | 55189 | 56529 | 446 | DNA topoisomerase II medium subunit [*Salmonella* phage PhiSH19] | 0.0 | 376/446(84%) | [YP_007007991.1](http://www.ncbi.nlm.nih.gov/protein/422937789?report=genbank&log$=prottop&blast_rank=1&RID=G1F6Y27H016) |
| 62 | - | 56522 | 58432 | 636 | topoisomerase II large subunit [*Shigella* phage phiSboM-AG3] | 0.0 | 550/637(86%) | [YP_003358499.1](http://www.ncbi.nlm.nih.gov/protein/282599187?report=genbank&log$=prottop&blast_rank=2&RID=G1F6Y27H016) |
| 63 | - | 58481 | 59059 | 192 | hypothetical protein [*Escherichia* phage ECML-4] | 3e-112 | 153/192(80%) | [AFO10307.1](http://www.ncbi.nlm.nih.gov/protein/397133798?report=genbank&log$=prottop&blast_rank=1&RID=G1F6Y27H016) |
| 64 | - | 59056 | 59541 | 161 | unnamed protein product [*Salmonella* phage SFP10] | 1e-108 | 152/161(94%) | [YP_004895180.1](http://www.ncbi.nlm.nih.gov/protein/351518452?report=genbank&log$=prottop&blast_rank=1&RID=G1F6Y27H016) |
| 65 | - | 59823 | 60404 | 193 | putative histone-like protein [*Serratia* phage phiMAM1] | 8e-18 | 48/175(27%) | [YP_007349173.1](http://www.ncbi.nlm.nih.gov/protein/440789463?report=genbank&log$=prottop&blast_rank=1&RID=G1F6Y27H016) |
| 66 | - | 60617 | 61426 | 269 | tail fiber protein [*Salmonella* phage STML-13-1] | 1e-163 | 228/269(85%) | [AFU64314.1](http://www.ncbi.nlm.nih.gov/protein/408387308?report=genbank&log$=prottop&blast_rank=2&RID=G1YCNCMX01R) |
| 67 | - | 61405 | 61797 | 130 | unnamed protein product [*Salmonella* phage SFP10] | 7e-87 | 125/130(96%) | [YP_004895175.1](http://www.ncbi.nlm.nih.gov/protein/351518447?report=genbank&log$=prottop&blast_rank=1&RID=G1YCNCMX01R) |
| 68 | - | 61829 | 62296 | 155 | unnamed protein product [*Salmonella* phage SFP10] | 2e-68 | 107/156(69%) | [YP_004895174.1](http://www.ncbi.nlm.nih.gov/protein/351518446?report=genbank&log$=prottop&blast_rank=1&RID=G1YCNCMX01R) |
| 69 | - | 62566 | 64152 | 528 | RIIB Protector from prophage-induced early lysis [*Salmonella* phage Vi01] | 0.0 | 388/537(72%) | [YP_004327373.1](http://www.ncbi.nlm.nih.gov/protein/326804502?report=genbank&log$=prottop&blast_rank=1&RID=G1YCNCMX01R) |
| 70 | - | 64186 | 66870 | 894 | RIIA protector from prophage-induced early lysis [*Salmonella* phage Vi01] | 0.0 | 538/896(60%) | [YP_004327372.1](http://www.ncbi.nlm.nih.gov/protein/326804501?report=genbank&log$=prottop&blast_rank=2&RID=G1YCNCMX01R) |
| 71 | - | 67045 | 67383 | 112 | unnamed protein product [*Salmonella* phage SFP10] | 6e-52 | 81/112(72%) | [YP_004895372.1](http://www.ncbi.nlm.nih.gov/protein/351518644?report=genbank&log$=prottop&blast_rank=1&RID=G3SFZF5F013) |
| 72 | - | 67755 | 68411 | 218 | unnamed protein product [*Salmonella* phage SFP10] | 2e-51 | 90/157(57%) | [YP_004895370.1](http://www.ncbi.nlm.nih.gov/protein/351518642?report=genbank&log$=prottop&blast_rank=1&RID=GMB4T8BP015) |
| 73 | - | 68408 | 68794 | 128 | hypothetical protein [*Dickeya* phage vB_DsoM_LIMEstone1] | 5e-14 | 47/125(38%) | [CCD57758.1](http://www.ncbi.nlm.nih.gov/protein/384228794?report=genbank&log$=prottop&blast_rank=1&RID=GMB4T8BP015) |
| 74 | - | 68860 | 69219 | 119 | hypothetical protein [*Dickeya* phage vB_DsoM_LIMEstone1] | 2e-39 | 65/114(57%) | [CCD57757.1](http://www.ncbi.nlm.nih.gov/protein/384228793?report=genbank&log$=prottop&blast_rank=1&RID=GMB4T8BP015) |
| 75 | - | 69239 | 69559 | 106 | n/a |  |  |  |
| 76 | - | 69562 | 70125 | 187 | hypothetical protein [*Shigella* phage phiSboM-AG3] | 3e-104 | 145/186(78%) | [YP_003358696.1](http://www.ncbi.nlm.nih.gov/protein/282599384?report=genbank&log$=prottop&blast_rank=1&RID=GMB4T8BP015) |
| 77 | - | 70142 | 71236 | 364 | hypothetical protein [*Salmonella* phage STML-13-1] | 0.0 | 263/369(71%) | [AFU64299.1](http://www.ncbi.nlm.nih.gov/protein/408387293?report=genbank&log$=prottop&blast_rank=1&RID=GMB4T8BP015) |
| 78 | - | 71547 | 72764 | 405 | hypothetical protein [*Escherichia* phage PhaxI] | 0.0 | 367/411(89%) | [YP_007002837.1](http://www.ncbi.nlm.nih.gov/protein/418488807?report=genbank&log$=prottop&blast_rank=1&RID=GMB4T8BP015) |
| 79 | - | 72768 | 73148 | 126 | orf245 gene product [*Escherichia* phage vB_EcoM_CBA120] | 2e-47 | 69/108(64%) | [YP_004957894.1](http://www.ncbi.nlm.nih.gov/protein/372199558?report=genbank&log$=prottop&blast_rank=1&RID=GMB5MN4V015) |
| 80 | - | 73351 | 74235 | 294 | putative uncharacterized protein [*Salmonella* phage PhiSH19] | 4e-157 | 219/292(75%) | [YP_007008137.1](http://www.ncbi.nlm.nih.gov/protein/422937935?report=genbank&log$=prottop&blast_rank=1&RID=GMB5MN4V015) |
| 81 | - | 74545 | 75336 | 263 | putative 5'(3') deoxyribonucleotidase [*Dickeya* phage vB_DsoM_LIMEstone1] | 2e-135 | 186/260(72%) | [CCD57745.1](http://www.ncbi.nlm.nih.gov/protein/384228781?report=genbank&log$=prottop&blast_rank=1&RID=GMB5MN4V015) |
| 82 | - | 75333 | 75665 | 110 | hypothetical protein [*Dickeya* phage vB_DsoM_LIMEstone1] | 1e-48 | 80/113(71%) | [CCD57744.1](http://www.ncbi.nlm.nih.gov/protein/384228780?report=genbank&log$=prottop&blast_rank=1&RID=GMB6Z6BV013) |
| 83 | - | 75732 | 78797 | 1021 | DNA polymerase [*Salmonella* phage SKML-39] | 0.0 | 882/997(88%) | [YP_007236285.1](http://www.ncbi.nlm.nih.gov/protein/431809389?report=genbank&log$=prottop&blast_rank=1&RID=GMB6Z6BV013) |
| 84 | + | 78807 | 79379 | 190 | putative uncharacterised protein [*Salmonella* phage Vi01] | 7e-127 | 178/190(94%) | [YP_004327560.1](http://www.ncbi.nlm.nih.gov/protein/326804689?report=genbank&log$=prottop&blast_rank=1&RID=GMB6Z6BV013) |
| 85 | + | 79727 | 80284 | 185 | hypothetical protein [*Escherichia* phage ECML-4] | 3e-123 | 170/185(92%) | [AFO10337.1](http://www.ncbi.nlm.nih.gov/protein/397133828?report=genbank&log$=prottop&blast_rank=1&RID=GMB6Z6BV013) |
| 86 | - | 80366 | 80818 | 150 | hypothetical protein [*Salmonella* phage PhiSH19] | 4e-84 | 126/150(84%) | [YP_007008128.1](http://www.ncbi.nlm.nih.gov/protein/422937926?report=genbank&log$=prottop&blast_rank=1&RID=GMB6Z6BV013) |
| 87 | - | 80851 | 81309 | 152 | unknown structural protein [*Dickeya* phage vB_DsoM_LIMEstone1] | 2e-70 | 99/150(66%) | [CCD57734.1](http://www.ncbi.nlm.nih.gov/protein/384228770?report=genbank&log$=prottop&blast_rank=1&RID=GMB6Z6BV013) |
| 88 | - | 81499 | 82704 | 401 | hypothetical protein [*Escherichia* phage ECML-4] | 0.0 | 352/401(88%) | [AFO10341.1](http://www.ncbi.nlm.nih.gov/protein/397133832?report=genbank&log$=prottop&blast_rank=1&RID=GMB6Z6BV013) |
| 89 | - | 82773 | 83405 | 210 | hypothetical protein [*Salmonella* phage STML-13-1] | 2e-56 | 105/236(44%) | [AFU64279.1](http://www.ncbi.nlm.nih.gov/protein/408387273?report=genbank&log$=prottop&blast_rank=1&RID=GMB6Z6BV013) |
| 90 | - | 86925 | 87485 | 186 | HNH endonuclease family protein [*Xanthomonas* phage OP1] | 8e-21 | 51/146(35%) | [YP_453589.1](http://www.ncbi.nlm.nih.gov/protein/84662624?report=genbank&log$=prottop&blast_rank=4&RID=GMJ65XG7013) |
| 91 | - | 88541 | 89149 | 202 | putative uncharacterized protein [*Salmonella* phage PhiSH19] | 4e-93 | 129/189(68%) | [YP_007008123.1](http://www.ncbi.nlm.nih.gov/protein/422937921?report=genbank&log$=prottop&blast_rank=1&RID=GMJ65XG7013) |
| 92 | + | 89465 | 91246 | 593 | baseplate wedge subunit [*Salmonella* phage Vi01] | 0.0 | 482/593(81%) | [YP_004327547.1](http://www.ncbi.nlm.nih.gov/protein/326804676?report=genbank&log$=prottop&blast_rank=2&RID=GMJ65XG7013) |
| 93 | + | 91230 | 92081 | 283 | putative baseplate wedge subunit gp7 [*Dickeya* phage vB_DsoM_LIMEstone1] | 5e-162 | 216/284(76%) | [CCD57723.1](http://www.ncbi.nlm.nih.gov/protein/384228759?report=genbank&log$=prottop&blast_rank=3&RID=GMJ65XG7013) |
| 94 | + | 92086 | 93411 | 441 | hypothetical protein [*Shigella* phage phiSboM-AG3] | 6e-135 | 229/443(52%) | [YP_003358666.1](http://www.ncbi.nlm.nih.gov/protein/282599354?report=genbank&log$=prottop&blast_rank=1&RID=GMJ70J0R013) |
| 95 | + | 93463 | 96681 | 1072 | putative tailspike protein [*Dickeya* phage vB_DsoM_LIMEstone1] | 3e-171 | 267/423(63%) | [CCD57721.1](http://www.ncbi.nlm.nih.gov/protein/384228757?report=genbank&log$=prottop&blast_rank=3&RID=GMJ70J0R013) |
| 96 | + | 96969 | 100706 | 1245 | putative tail fiber protein [*Escherichia* phage PhaxI];  tailspike protein [*Shigella* phage phiSboM-AG3];  K5 lyase [*Enterobacteria* phage K5] | 1e-71  1e-70  5e-11 | 125/167(75%)  128/199(64%)  61/222(27%) | [YP_007002808.1](http://www.ncbi.nlm.nih.gov/protein/418488828?report=genbank&log$=prottop&blast_rank=1&RID=GMJ70J0R013)  [YP_003358663.1](http://www.ncbi.nlm.nih.gov/protein/282599351?report=genbank&log$=prottop&blast_rank=3&RID=GMJ70J0R013)  [CAA71133.2](http://www.ncbi.nlm.nih.gov/protein/298905496?report=genbank&log$=prottop&blast_rank=44&RID=GMJ70J0R013) |
| 97 | + | 100804 | 102777 | 657 | tail spike protein head-binding protein [*Serratia* phage phiMAM1];  hypothetical protein HMPREF1024_01923 [*Klebsiella* sp. 4_1_44FAA] | 1e-11  0.0 | 35/89(39%)  471/554(85%) | [YP_007349017.1](http://www.ncbi.nlm.nih.gov/protein/440789307?report=genbank&log$=prottop&blast_rank=8&RID=GMJ70J0R013)  [ZP_09345892.1](http://www.ncbi.nlm.nih.gov/protein/365139545?report=genbank&log$=prottop&blast_rank=1&RID=GMJ70J0R013) |
| 98 | + | 102790 | 104613 | 607 | hypothetical protein RaK2_00526 [*Enterobacteria* phage vB_KleM-RaK2] | 5e-63 | 185/588(31%) | [YP_007007681.1](http://www.ncbi.nlm.nih.gov/protein/422937414?report=genbank&log$=prottop&blast_rank=1&RID=GN5Y46SZ013) |
| 99 | + | 104626 | 106254 | 542 | hypothetical protein RaK2_00525 [*Enterobacteria* phage vB_KleM-RaK2] | 4e-65 | 172/568(30%) | [YP_007007680.1](http://www.ncbi.nlm.nih.gov/protein/422937413?report=genbank&log$=prottop&blast_rank=2&RID=GN5Y46SZ013) |
| 100 | + | 106583 | 111433 | 1616 | vrlC protein [*Escherichia* phage ECML-4] | 0.0 | 1362/1612(84%) | [AFO10354.1](http://www.ncbi.nlm.nih.gov/protein/397133845?report=genbank&log$=prottop&blast_rank=1&RID=GN5Y46SZ013) |
| 101 | + | 111716 | 112054 | 112 | unnamed protein product [*Salmonella* phage SFP10];  putative capsid protein [*Serratia* phage phiMAM1] | 3e-47  4e-22 | 74/104(71%)  43/96(45%) | [YP_004895328.1](http://www.ncbi.nlm.nih.gov/protein/351518600?report=genbank&log$=prottop&blast_rank=1&RID=GN5ZG8UM015)  [YP_007349025.1](http://www.ncbi.nlm.nih.gov/protein/440789315?report=genbank&log$=prottop&blast_rank=6&RID=GN5ZG8UM015) |
| 102 | + | 112041 | 112793 | 250 | Gp13 neck protein [*Salmonella* phage Vi01] | 8e-162 | 233/250(93%) | [YP_004327537.1](http://www.ncbi.nlm.nih.gov/protein/326804666?report=genbank&log$=prottop&blast_rank=1&RID=GN5ZG8UM015) |
| 103 | + | 113018 | 113737 | 239 | Gp14 neck protein [*Salmonella* phage Vi01] | 4e-143 | 195/213(92%) | [YP_004327535.1](http://www.ncbi.nlm.nih.gov/protein/326804664?report=genbank&log$=prottop&blast_rank=3&RID=GN5ZG8UM015) |
| 104 | + | 113740 | 114438 | 232 | Gp15 proximal tail sheath stabilization [*Salmonella* phage Vi01] | 5e-146 | 193/230(84%) | [YP_004327534.1](http://www.ncbi.nlm.nih.gov/protein/326804663?report=genbank&log$=prottop&blast_rank=1&RID=GN5ZG8UM015) |
| 105 | + | 114441 | 115127 | 228 | Gp16 terminase DNA packaging enzyme small subunit [*Salmonella* phage Vi01] | 8e-97 | 157/236(67%) | [YP_004327533.1](http://www.ncbi.nlm.nih.gov/protein/326804662?report=genbank&log$=prottop&blast_rank=7&RID=GP7G9J44013) |
| 106 | + | 115108 | 117318 | 736 | Gp17 terminase subunit for DNA packaging, nuclease and ATPase [*Salmonella* phage Vi01] | 0.0 | 647/736(88%) | [YP_004327532.1](http://www.ncbi.nlm.nih.gov/protein/326804661?report=genbank&log$=prottop&blast_rank=2&RID=GP7G9J44013) |
| 107 | + | 117371 | 119266 | 631 | Gp18 tail sheath protein [*Salmonella* phage Vi01] | 0.0 | 574/631(91%) | [YP_004327531.1](http://www.ncbi.nlm.nih.gov/protein/326804660?report=genbank&log$=prottop&blast_rank=2&RID=GP7G9J44013) |
| 108 | + | 119335 | 119790 | 151 | GIY-YIG endonuclease [*Salmonella* phage Vi01] | 9e-92 | 125/150(83%) | [YP_004327530.1](http://www.ncbi.nlm.nih.gov/protein/326804659?report=genbank&log$=prottop&blast_rank=1&RID=GP7G9J44013) |
| 109 | + | 119825 | 120358 | 177 | Gp19 tail tube protein [*Salmonella* phage Vi01] | 1e-123 | 171/177(97%) | [YP_004327529.1](http://www.ncbi.nlm.nih.gov/protein/326804658?report=genbank&log$=prottop&blast_rank=1&RID=GP7G9J44013) |
| 110 | + | 120427 | 122109 | 560 | Gp20 portal vertex protein of the head [*Salmonella* phage Vi01] | 0.0 | 447/539(83%) | [YP_004327528.1](http://www.ncbi.nlm.nih.gov/protein/326804657?report=genbank&log$=prottop&blast_rank=1&RID=GP7HBV9701R) |
| 111 | + | 122320 | 122637 | 105 | putative prohead core protein [*Dickeya* phage vB_DsoM_LIMEstone1] | 3e-58 | 91/101(90%) | [CCD57702.1](http://www.ncbi.nlm.nih.gov/protein/384228738?report=genbank&log$=prottop&blast_rank=2&RID=GP7HBV9701R) |
| 112 | + | 122645 | 123313 | 222 | putative prohead protease gp21 [*Dickeya* phage vB_DsoM_LIMEstone1] | 3e-143 | 194/222(87%) | [CCD57701.1](http://www.ncbi.nlm.nih.gov/protein/384228737?report=genbank&log$=prottop&blast_rank=1&RID=GP7HBV9701R) |
| 113 | + | 123359 | 124219 | 286 | Gp22 prohead core scaffold protein [*Salmonella* phage Vi01] | 3e-108 | 168/237(71%) | [YP_004327524.1](http://www.ncbi.nlm.nih.gov/protein/326804653?report=genbank&log$=prottop&blast_rank=5&RID=GP7HBV9701R) |
| 114 | + | 124305 | 125636 | 443 | phage major head protein/major capsid protein [*Salmonella* phage SFP10] | 0.0 | 410/440(93%) | AEN94237 |
| 115 | + | 126446 | 126748 | 100 | putative uncharacterised protein [*Salmonella* phage Vi01] | 7e-31 | 53/81(65%) | [YP_004327521.1](http://www.ncbi.nlm.nih.gov/protein/326804650?report=genbank&log$=prottop&blast_rank=1&RID=GPNT6ZS201R) |
| 116 | + | 126764 | 127204 | 146 | hypothetical protein [*Salmonella* phage STML-13-1] | 2e-51 | 81/141(57%) | [AFU64246.1](http://www.ncbi.nlm.nih.gov/protein/408387240?report=genbank&log$=prottop&blast_rank=1&RID=GPNT6ZS201R) |
| 117 | + | 127201 | 127644 | 147 | hypothetical protein [*Serratia* phage KSP90] | 2e-51 | 83/146(57%) | [BAH15173.1](http://www.ncbi.nlm.nih.gov/protein/221271418?report=genbank&log$=prottop&blast_rank=1&RID=GPNT6ZS201R) |
| 118 | + | 128627 | 129070 | 147 | hypothetical protein [*Shigella* phage phiSboM-AG3];  putative GTP-binding protein [*Deftia* phage phiW-14] | 5e-53  3e-13 | 90/146(62%)  47/133(35%) | [YP_003358636.1](http://www.ncbi.nlm.nih.gov/protein/282599324?report=genbank&log$=prottop&blast_rank=1&RID=GPNTRNHS015)  [YP_003359063.1](http://www.ncbi.nlm.nih.gov/protein/282599146?report=genbank&log$=prottop&blast_rank=12&RID=GPNTRNHS015) |
| 119 | + | 129155 | 130024 | 289 | hypothetical protein [*Shigella* phage phiSboM-AG3] | 3e-122 | 175/242(72%) | [YP_003358634.1](http://www.ncbi.nlm.nih.gov/protein/282599322?report=genbank&log$=prottop&blast_rank=1&RID=GPNTRNHS015) |
| 120 | - | 130025 | 130675 | 216 | hypothetical protein [*Escherichia* phage ECML-4] | 4e-114 | 153/213(72%) | [AFO10384.1](http://www.ncbi.nlm.nih.gov/protein/397133875?report=genbank&log$=prottop&blast_rank=1&RID=GPRWB9GD01R) |
| 121 | + | 130714 | 131214 | 166 | Gp3-tail completion [*Salmonella* phage Vi01] | 5e-102 | 143/164(87%) | [YP_004327511.1](http://www.ncbi.nlm.nih.gov/protein/326804640?report=genbank&log$=prottop&blast_rank=1&RID=GPRWB9GD01R) |
| 122 | + | 131252 | 131713 | 153 | putative DNA repair/recombination protein UvsY [*Shigella* phage phiSboM-AG3] | 3e-79 | 115/146(79%) | [YP_003358631.1](http://www.ncbi.nlm.nih.gov/protein/282599319?report=genbank&log$=prottop&blast_rank=4&RID=GPRWB9GD01R) |
| 123 | + | 131716 | 132462 | 248 | putative exonuclease [*Dickeya* phage vB_DsoM_LIMEstone1] | 1e-150 | 200/248(81%) | [CCD57686.1](http://www.ncbi.nlm.nih.gov/protein/384228722?report=genbank&log$=prottop&blast_rank=7&RID=GPRWB9GD01R) |
| 124 | + | 132490 | 134007 | 505 | putative type III restriction enzyme [*Salmonella* phage Vi01] | 0.0 | 399/501(80%) | [YP_004327508.1](http://www.ncbi.nlm.nih.gov/protein/326804637?report=genbank&log$=prottop&blast_rank=2&RID=GPRWB9GD01R) |
| 125 | - | 133992 | 134381 | 129 | hypothetical protein [*Dickeya* phage vB_DsoM_LIMEstone1] | 5e-14 | 46/126(37%) | [CCD57682.1](http://www.ncbi.nlm.nih.gov/protein/384228718?report=genbank&log$=prottop&blast_rank=1&RID=GPRWT1ZS01R) |
| 126 | + | 134696 | 135364 | 222 | Gp45-sliding clmap holder [*Salmonella* phage Vi01] | 3e-149 | 202/222(91%) | [YP_004327506.1](http://www.ncbi.nlm.nih.gov/protein/326804635?report=genbank&log$=prottop&blast_rank=1&RID=GPRWT1ZS01R) |
| 127 | + | 135450 | 136439 | 329 | Gp44-sliding clamp holder [*Salmonella* phage Vi01] | 0.0 | 256/328(78%) | [YP_004327505.1](http://www.ncbi.nlm.nih.gov/protein/326804634?report=genbank&log$=prottop&blast_rank=2&RID=GPRWT1ZS01R) |
| 128 | + | 136430 | 136864 | 144 | clamp holder for DNA polymerase [*Salmonella* phage Vi01] | 2e-79 | 109/140(78%) | [YP_004327504.1](http://www.ncbi.nlm.nih.gov/protein/326804633?report=genbank&log$=prottop&blast_rank=2&RID=GPRWT1ZS01R) |
| 129 | + | 136893 | 137357 | 154 | RegA translational repressor protein [*Shigella* phage phiSboM-AG3] | 5e-99 | 134/154(87%) | [YP_003358624.1](http://www.ncbi.nlm.nih.gov/protein/282599312?report=genbank&log$=prottop&blast_rank=3&RID=GPRWT1ZS01R) |
| 130 | + | 137374 | 138240 | 288 | conserved uncharacterised protein [*Salmonella* phage Vi01] | 6e-155 | 217/291(75%) | [YP_004327502.1](http://www.ncbi.nlm.nih.gov/protein/326804631?report=genbank&log$=prottop&blast_rank=1&RID=GRX9FHE5013) |
| 131 | + | 138312 | 139532 | 406 | conserved uncharacterised protein [*Salmonella* phage Vi01] | 0.0 | 297/406(73%) | [YP_004327501.1](http://www.ncbi.nlm.nih.gov/protein/326804630?report=genbank&log$=prottop&blast_rank=1&RID=GRX9FHE5013) |
| 132 | + | 139870 | 141906 | 678 | hypothetical protein [*Escherichia* phage ECML-4] | 0.0 | 471/678(69%) | [AFO10396.1](http://www.ncbi.nlm.nih.gov/protein/397133887?report=genbank&log$=prottop&blast_rank=1&RID=GRX9FHE5013) |
| 133 | + | 141943 | 142317 | 124 | hypothetical protein [*Salmonella* phage SKML-39] | 2e-55 | 92/124(74%) | [YP_007236140.1](http://www.ncbi.nlm.nih.gov/protein/431809244?report=genbank&log$=prottop&blast_rank=1&RID=GRX9FHE5013) |
| 134 | + | 142416 | 143270 | 284 | ribose-phosphate pyrophosphokinase [*Salmonella* phage SKML-39] | 0.0 | 274/284(96%) | [YP_007236143.1](http://www.ncbi.nlm.nih.gov/protein/431809247?report=genbank&log$=prottop&blast_rank=1&RID=GRX9FHE5013) |
| 135 | + | 143267 | 144919 | 550 | nicotinamide phosphoribosyltransferase [*Salmonella* phage SKML-39] | 0.0 | 506/554(91%) | [YP_007236144.1](http://www.ncbi.nlm.nih.gov/protein/431809248?report=genbank&log$=prottop&blast_rank=1&RID=GRXZ94MT013) |
| 136 | + | 145006 | 145383 | 125 | hypothetical protein SPM24T3_14050 [*Serratia* sp. M24T3] | 6e-49 | 71/120(59%) | [ZP_09970895.1](http://www.ncbi.nlm.nih.gov/protein/383815483?report=genbank&log$=prottop&blast_rank=1&RID=GRXZ94MT013) |
| 137 | + | 145384 | 146139 | 251 | hypothetical protein [*Salmonella* phage SKML-39] | 2e-74 | 122/252(48%) | [YP_007236142.1](http://www.ncbi.nlm.nih.gov/protein/431809246?report=genbank&log$=prottop&blast_rank=1&RID=GRXZ94MT013) |
| 138 | + | 146204 | 146560 | 118 | putative uncharacterized protein [*Salmonella* phage PhiSH19] | 3e-48 | 78/118(66%) | [YP_007008078.1](http://www.ncbi.nlm.nih.gov/protein/422937876?report=genbank&log$=prottop&blast_rank=1&RID=GRXZ94MT013) |
| 139 | + | 146704 | 148902 | 732 | putative uncharacterised protein [*Salmonella* phage Vi01] | 0.0 | 566/738(77%) | [YP_004327494.1](http://www.ncbi.nlm.nih.gov/protein/326804623?report=genbank&log$=prottop&blast_rank=1&RID=GRXZ94MT013) |
| 140 | + | 148947 | 149270 | 107 | putative acyl carrier protein [*Dickeya* phage vB_DsoM_LIMEstone1] | 2e-36 | 62/107(58%) | [CCD57668.1](http://www.ncbi.nlm.nih.gov/protein/384228704?report=genbank&log$=prottop&blast_rank=5&RID=GTC9TB6N01R) |
| 141 | + | 149897 | 150265 | 122 | hypothetical protein [*Salmonella* phage SKML-39] | 6e-34 | 63/125(50%) | [YP_007236150.1](http://www.ncbi.nlm.nih.gov/protein/431809254?report=genbank&log$=prottop&blast_rank=1&RID=GTCA8RE101R) |
| 142 | + | 150262 | 150570 | 102 | hypothetical protein [*Salmonella* phage SKML-39] | 3e-07 | 27/86(31%) | [YP_007236151.1](http://www.ncbi.nlm.nih.gov/protein/431809255?report=genbank&log$=prottop&blast_rank=1&RID=GTCA8RE101R) |
| 143 | + | 150864 | 151313 | 149 | phage assiociated protein [*Salmonella* phage Vi01] | 3e-98 | 138/149(93%) | [YP_004327488.1](http://www.ncbi.nlm.nih.gov/protein/326804617?report=genbank&log$=prottop&blast_rank=1&RID=GRYS4H5S01R) |
| 144 | + | 151392 | 152078 | 228 | putative uncharacterised protein [*Salmonella* phage Vi01] | 9e-143 | 196/228(86%) | [YP_004327487.1](http://www.ncbi.nlm.nih.gov/protein/326804616?report=genbank&log$=prottop&blast_rank=1&RID=GRYS4H5S01R) |
| 145 | + | 152075 | 152422 | 115 | putative uncharacterized protein [*Salmonella* phage PhiSH19] | 2e-46 | 75/106(71%) | [YP_007008072.1](http://www.ncbi.nlm.nih.gov/protein/422937870?report=genbank&log$=prottop&blast_rank=1&RID=GTCW1NVF01R) |
| 146 | + | 152473 | 153213 | 246 | hypothetical protein [*Salmonella* phage STML-13-1] | 2e-46 | 101/259(39%) | [AFU64210.1](http://www.ncbi.nlm.nih.gov/protein/408387204?report=genbank&log$=prottop&blast_rank=1&RID=GRYS4H5S01R) |
| 147 | + | 153262 | 153969 | 235 | ImpD [*Salmonella* phage PhiSH19] | 2e-132 | 175/221(79%) | [YP_007008070.1](http://www.ncbi.nlm.nih.gov/protein/422937868?report=genbank&log$=prottop&blast_rank=2&RID=GRYS4H5S01R) |
| 148 | + | 154222 | 154545 | 107 | superinfection exclusion protein [*Salmonella* phage PhiSH19] | 3e-55 | 81/106(76%) | [YP_007008068.1](http://www.ncbi.nlm.nih.gov/protein/422937866?report=genbank&log$=prottop&blast_rank=3&RID=GTCBY31H01R) |
| 149 | - | 154550 | 155053 | 167 | n/a |  |  |  |
| 150 | + | 155044 | 156765 | 573 | ATP-dependent helicase [*Salmonella* phage Vi01] | 0.0 | 481/573(84%) | [YP_004327477.1](http://www.ncbi.nlm.nih.gov/protein/326804606?report=genbank&log$=prottop&blast_rank=2&RID=GTCBY31H01R) |
| 151 | - | 156762 | 157529 | 255 | unknown structural protein [*Dickeya* phage vB_DsoM_LIMEstone1] | 7e-152 | 204/255(80%) | [CCD57654.1](http://www.ncbi.nlm.nih.gov/protein/384228690?report=genbank&log$=prottop&blast_rank=1&RID=GTCBY31H01R) |
| 152 | + | 157571 | 158110 | 179 | ribonuclease HI [*Escherichia* phage ECML-4] | 1e-107 | 153/176(87%) | [AFO10419.1](http://www.ncbi.nlm.nih.gov/protein/397133910?report=genbank&log$=prottop&blast_rank=1&RID=GTCBY31H01R) |
| 153 | + | 158114 | 158884 | 256 | Gp55 sigma factor for late transcription [*Salmonella* phage Vi01] | 1e-158 | 209/256(82%) | [YP_004327474.1](http://www.ncbi.nlm.nih.gov/protein/326804603?report=genbank&log$=prottop&blast_rank=1&RID=GTCBY31H01R) |
| 154 | + | 158871 | 159989 | 372 | Gp47 recombination protein subunit [*Salmonella* phage Vi01] | 0.0 | 325/372(87%) | [YP_004327473.1](http://www.ncbi.nlm.nih.gov/protein/326804602?report=genbank&log$=prottop&blast_rank=1&RID=GTCBY31H01R) |

Note:

*a*. ORFs >100 codons, predicted by the GeneMark program. *b*. (+) ORFs in clockwise, and (-) in a counterclockwise orientation. *c.* Determined by BLAST-P (non-redundant ).
